# Ni(I)–Alkyl Complexes Bearing Phenanthroline Ligands: Experimental Evidence for CO_2_ Insertion at Ni(I) Centers

**DOI:** 10.1021/jacs.0c04695

**Published:** 2020-06-10

**Authors:** Rosie J. Somerville, Carlota Odena, Marc F. Obst, Nilay Hazari, Kathrin H. Hopmann, Ruben Martin

**Affiliations:** † Institute of Chemical Research of Catalonia (ICIQ), The Barcelona Institute of Science and Technology, Av. Països Catalans 16, 43007 Tarragona, Spain; ‡ Departament de Química Analítica i Química Orgànica, Universitat Rovira i Virgili, c/Marcel·lí Domingo 1, 43007 Tarragona, Spain; § ICREA, Passeig Lluís Companys 23, 08010 Barcelona, Spain; ∥ Hylleraas Center for Quantum Molecular Sciences, Department of Chemistry, UiT The Arctic University of Norway, N-9307 Tromsø, Norway; ⊥ Department of Chemistry, Yale University, P.O. Box 208107, New Haven, Connecticut 06520, United States

## Abstract

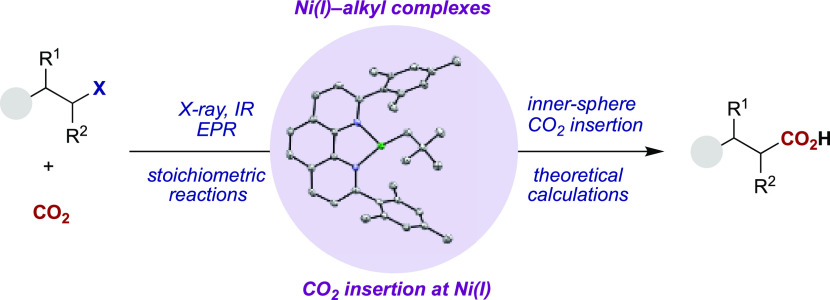

Although the catalytic carboxylation of unactivated alkyl electrophiles has reached remarkable levels of sophistication, the intermediacy of (phenanthroline)Ni(I)–alkyl species—complexes proposed in numerous Ni-catalyzed reductive cross-coupling reactions—has been subject to speculation. Herein we report the synthesis of such elusive (phenanthroline)Ni(I) species and their reactivity with CO_2_, allowing us to address a long-standing question related to Ni-catalyzed carboxylation reactions.

Over the past decade, Ni-catalyzed reductive carboxylation reactions involving organic (pseudo)halides and carbon dioxide have received considerable attention as methodologies for the preparation of many synthetically useful carboxylic acids.^1^ Among the wide variety of Ni-catalyzed reductive carboxylation reactions developed to date, the carboxylation of *unactivated* alkyl (pseudo)halides possessing β-hydrogens was found to be particularly challenging.^2^ This is likely due to the propensity of the alkylnickel intermediates that are formed via C(sp^3^)–X scission (X = Br, Cl, OSO_2_R) to undergo unproductive reduction, β-hydride elimination, and homocoupling reactions.^3^ Although nickel catalysts supported by (di)phosphine or N-heterocyclic carbene ligands are routinely employed in a myriad of Ni-catalyzed C–C and C–heteroatom bond-forming reactions,^4^ only finely tuned 1,10-phenanthroline derivatives—phen ligands—have enabled the carboxylation of unactivated alkyl electrophiles either at the initial C(sp^3^)–X site or at remote C(sp^3^)–H bonds via chain-walking of the Ni catalyst along the alkyl side chain (Scheme 1).^2,5^ Furthermore, a careful analysis of the literature indicates that phen ligands are also crucial for a wide number of Ni-catalyzed cross-couplings of unactivated alkyl halides, indicating that the importance of these ligands extends beyond carboxylation reactions.^4,6^

**Scheme 1:**
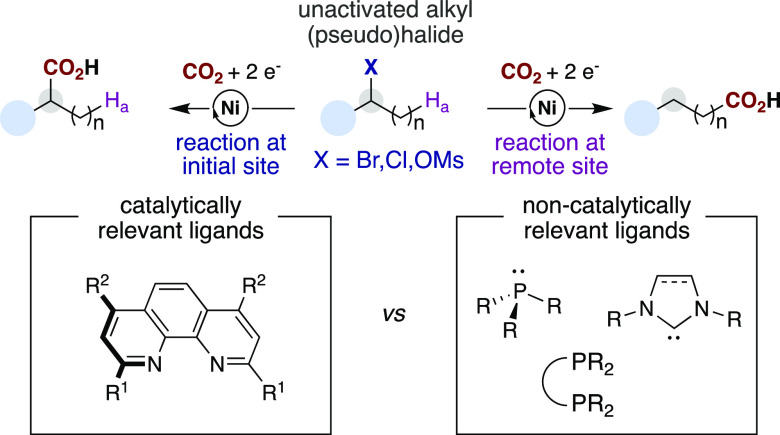
Carboxylation of Unactivated Alkyl Electrophiles

Despite significant advances in methodology design, the mechanism of the Ni-catalyzed reductive carboxylation of unactivated alkyl (pseudo)halides with CO_2_ is poorly understood. At present, our knowledge is primarily based on studies using *aryl* (pseudo)halide substrates. These suggest that CO_2_ insertion at a (phen)Ni(I)–alkyl complex is a crucial elementary step (Scheme 2, left).^7,8^ However, it is worth noting that no (phen)Ni(I)–alkyl complexes have been structurally characterized or even observed spectroscopically, probably because of the fleeting nature and high reactivity of these paramagnetic species.^9^ Elegant efforts toward this goal were recently described by Diao, and cultimated in the synthesis of (diphosphine)Ni(I)–alkyl complexes and investigations into their reactivity with CO_2_.^10,11^ Unfortunately, diphosphine ligands have not been shown to facilitate the Ni-catalyzed carboxylation of unactivated alkyl (pseudo)halides (Scheme 1).^2,12^ Therefore, a study aimed at preparing well-defined Ni(I)–alkyl complexes bearing catalytically relevant phen ligands would represent (a) an opportunity to study the reactivity of elusive Ni(I)–alkyl complexes supported by nitrogen-donor ligands, (b) a foundation for investigating the mechanistic intricacies of catalytic reductive carboxylation reactions, and (c) a starting point for understanding the speciation of Ni catalysts supported by phen ligands in related cross-coupling and chain-walking reactions.^4^ Herein we report the realization of these goals through the synthesis and isolation of Ni(I)–alkyl complexes bearing phen ligands, which has enabled us to obtain experimental evidence for rapid CO_2_ insertion at Ni(I)–carbon bonds (Scheme 2, right). These results not only shed light on a long-speculated mechanistic step but also support efforts to exploit and expand the reactivity of (phen)Ni(I)–alkyl intermediates through photoredox or electrochemical methodologies.^2a,2b,4,13^

**Scheme 2:**
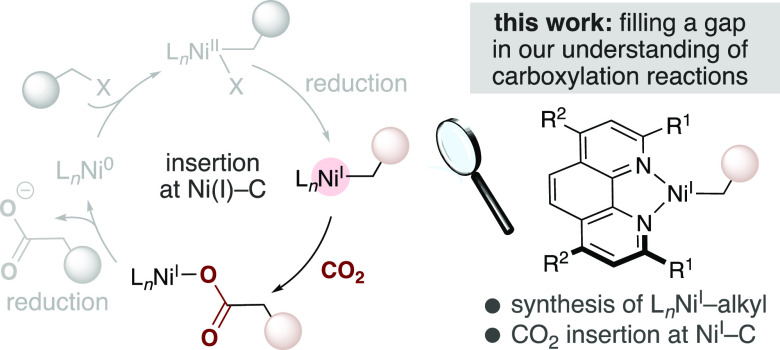
Proposed Reductive Carboxylation Mechanism via CO_2_ Insertion at Phen-Ligated Ni(I)–Alkyl Species

Our study began by establishing a route to Ni(I)–halide complexes bearing phen ligands **L1** or **L2**. The choice of these ligands was not arbitrary, as substituents adjacent to the nitrogen donor atoms are critical in Ni-catalyzed reductive carboxylation reactions of unactivated alkyl (pseudo)halides.^2^ Steric shielding by the bulky mesityl substituents of **L1** may help to stabilize our targeted Ni(I)–alkyl complexes, which are likely highly reactive.^9,14^ Additionally, **L2** is employed in the Ni-catalyzed chain-walking carboxylation of alkyl bromides.^2c^ We envisioned that (**L**)Ni(I)–alkyl species could be accessed by alkylation of inner-sphere Ni(I)–halide complexes with an appropriate organometallic reagent. However, at the outset of our investigations, it was unclear whether an inner-sphere (**L**)Ni(I)–halide precursor could be obtained, as the most closely related reported species bearing a phen ligand was the outer-sphere halide complex [Ni(**L**)_2_]Cl, formed via oxidation of Ni(0)**L**_2_ (**L** = 2,9-dimethylphen) with AgCl.^8,14,16^ In order to avoid the synthesis of Ni(0)**L**_2_ complexes and the purification steps required to remove oxidation byproducts, we hypothesized that inner sphere (**L**)Ni(I)X (X = Br, Cl) might be obtained via comproportionation of (**L**)NiX_2_ with [Ni(COD)_2_] in the presence of 1 equiv of bulky **L**.^15,16^ This was indeed the case, and deep-blue (**L**)Ni(I)X species were obtained in high yields (Figure 1, left). The presence of the inner-sphere halide ligand was confirmed by X-ray crystallographic analysis of **1-Cl** and **2-Cl**. In addition, the axial electron paramagnetic resonance (EPR) spectra of the four (**L**)Ni(I)X complexes at 77 K support the presence of a Ni-centered radical. These results are noteworthy, as they represent examples of Ni(I) complexes bearing phen ligands with the halide directly coordinated to the Ni center.^16,17^ With a reliable route to (**L1,L2**)Ni(I)X in hand, we turned our attention to accessing the targeted Ni(I)–alkyl complexes via alkylation. An initial survey of the stability of the resulting Ni(I)–alkyl products was carried out by monitoring these reactions using EPR spectroscopy. As expected, the choice of alkyl group, reaction temperature, and ligand employed all influenced the reaction outcome. For example, reactions with EtMgBr and MeMgCl resulted in negligible amounts of new metal-centered radicals, if any. Analysis of these reactions by ^1^H NMR spectroscopy indicated the presence of Ni(0)**L**_*n*_ complexes, suggesting decomposition pathways arising from β-hydride elimination, reduction, and/or homolytic cleavage.^18^

**Figure 1:**
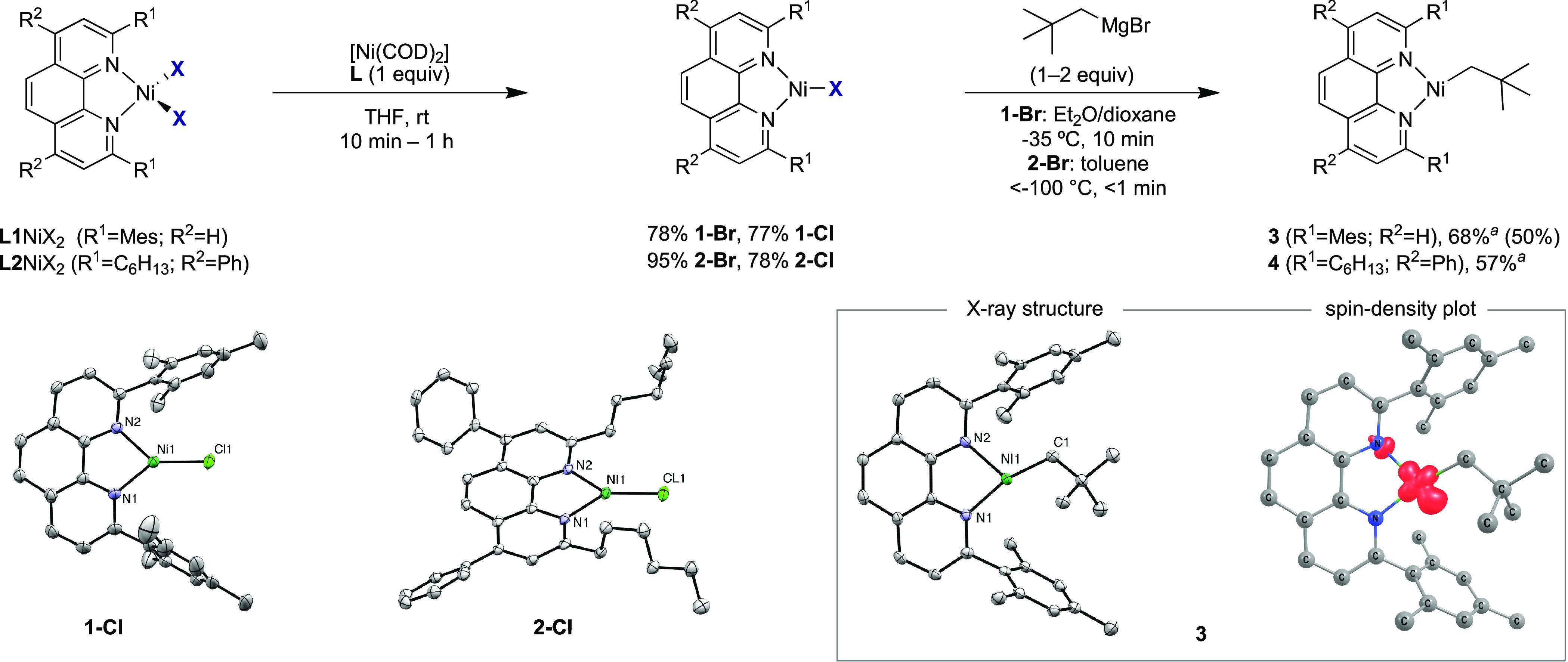
(top) Synthesis of Ni(I)–X and Ni(I)–alkyl complexes. ^*a*^Yield determined by EPR spectroscopy against Cu(II) standards. All other yields are isolated yields (0.010 mmol scale for **3**). (bottom left and center) X-ray structures with thermal ellipsoids drawn at the 50% probability level (see the Supporting Information for details). Selected distances (Å) and angles (deg): **1-Cl**: Ni1–Cl1 2.1064(6), N1–Ni1–Cl1 140.24(6), N2–Ni1–Cl1 136.32(6). **2-Cl**: Ni–Cl1 2.1417(9), N1–Ni–Cl1 133.80(8), N2–Ni–Cl1 142.61(9). **3**: Ni–C1 1.961(3), N1–Ni–C1 156.74(14), C1–Ni–N2 114.25(13). (bottom right) Calculated spin-density plot of **3** with a spin population of 0.94 on Ni (PBE-D3BJ/def2-TZVP, isovalue = 0.01; Figure S33).

Gratifyingly, the reactions of **1-Br** and **2-Br** with neopentylMgBr resulted in new rhombic EPR spectra, suggesting that the desired alkylation may have taken place.^19^ Low-temperature crystallization (−35 °C, Et_2_O/pentane) furnished deep-green crystals suitable for X-ray diffraction, allowing us to identify three-coordinate [(**L1**)Ni(I)CH_2_*t*Bu] (**3**) (Figure 1, right). Density functional theory (DFT) calculations support the Ni(I) description, with one unpaired electron centered on Ni (Figures 1 and S33). The synthesis of **3** is particularly noteworthy: to the best of our knowledge, *it is the first Ni(I)–alkyl complex to be obtained with a catalytically relevant phen ligand*. The Ni–C bond distance of 1.961(3) Å is similar to that of Ni(I) complexes bearing phosphine or NHC ligands.^10,20^ The Ni coordination plane is offset by ca. 23° from the mean plane through **L1**, presumably because of the steric bulk of the neopentyl ligand. Interestingly, the N–Ni–C angles in **3** are 114.25(13)° and 156.74(14)°. The distortion of **3** to this T-shaped geometry is similar to that observed in a related diphosphine species [(dtbpe)Ni(CH_2_*t*Bu)] (dtbpe = 1,2-bis(di-*tert*-butylphosphino)ethane) (110.97(8)° and 157.82(8)°).^20a^ This geometry is electronically favored for a range of three-coordinate Ni(I) complexes and differs from the Y-shaped geometry of **1-Cl** and **2-Cl**.^21,22^ We propose that the geometry of the latter complexes is due to the π-donating nature of the chloride ligand, which has been shown to favor Y-shaped complexes.^22^ Alkylation of **2-Br** at low temperature gave [(**L2**)Ni(I)CH_2_*t*Bu] (**4**) in 57% yield as estimated by EPR spectroscopy against a Cu(II) standard. Unfortunately, the thermal instability of **4** prevented its isolation or characterization by X-ray diffraction.

Next, we turned our attention to an investigation of CO_2_ insertion into the Ni(I)–C bond en route to Ni(I) carboxylate complexes, proposed to be the key elementary step in the catalytic carboxylation of alkyl (pseudo)halides (Figure 2).^7,8^ Prior to these insertion experiments, however, an anion metathesis reaction between **1-Cl** and *t*BuCH_2_CO_2_K was performed to obtain reference EPR and IR spectra of the proposed CO_2_ insertion product (Figure 2, left). Gratifyingly, spectroscopic analysis of the reaction mixture showed the formation of a complex distinct from both **3** and **1-Cl** and supported the formation of Ni(I)–carboxylate complex **5**. For example, the band in the IR spectrum at 1543 cm^–1^ is suggestive of a ν_asym_ carboxylate stretch.^23^ Furthermore, although repeated attempts to crystallize **5** did not provide crystals suitable for X-ray diffraction, the observed stretching frequency combined with the absence of signals between 1200 and 1400 cm^–1^ suggests κ^2^ coordination of the carboxylate to the Ni(I) center.^23a^ This was supported by DFT calculations that suggested a pseudotetrahedral geometry for **5** (Figure 3, right) with a computed stretching frequency of 1484 cm^–1^ (Figure S36). With these results in hand, we next investigated the reaction between **3** and CO_2_ (1 bar) at −60 °C (Figure 2, right). Analysis by EPR spectroscopy (77 K) showed the disappearance of the rhombic signal of **3** and the appearance of a new pseudoaxial signal with *g*_*x*_, *g*_*y*_ > *g*_*z*_ that very closely resembles the spectrum of **5** (Figure 2, center). Importantly, comparison of the IR spectrum of the product of direct CO_2_ insertion with that of the anion metathesis product showed an identical ν_asym_ carboxylate stretch at 1543 cm^–1^. Particularly illustrative was the disappearance of this signal and the appearance of new signals at lower wavenumber when the reaction was performed with ^13^CO_2_, providing evidence that **5** was formed via CO_2_ insertion into the Ni(I)–C bond. Calculations predicted a 34 cm^–1^ shift to lower wavenumbers upon incorporation of ^13^C, consistent with the observed shift of 38 cm^–1^ to a band at 1505 cm^–1^ (Figures S18 and S37). Insertion was also corroborated indirectly by quenching in situ-generated **5** with dilute HCl and observing a 52% yield of *tert*-butylacetic acid (Scheme 3, top). These observations are consistent with DFT calculations indicating facile CO_2_ insertion into the Ni(I)–C bond of **3** with a free energy barrier of 7.7 kcal mol^–1^ relative to **3** and free CO_2_ (Figure 3, middle). The calculations argue against the formation of a stable Ni–CO_2_ adduct before insertion (Figure S38), with interactions between Ni and CO_2_ first becoming significant at the carboxylation transition state (TS), where CO_2_ is significantly bent (137°) and interacts with Ni in a η^2^(C,O) fashion (Figure 3, center). Notably, an alternative *outer-sphere* insertion where CO_2_ does not interact with Ni in the transition state is predicted to have a barrier of 22.7 kcal mol^–1^, a 15.0 kcal mol^–1^ penalty compared to the inner-sphere pathway (Figure 3, center).^24^ Although an inner-sphere pathway has been calculated for the Ni/PCp_3_-catalyzed reductive carboxylation of benzyl halides,^25^ our data contrast with the outer-sphere pathway suggested for (Xantphos)Ni(I)–methyl and (PCP pincer)Ni(II)–methyl complexes.^9,11^ While one might argue that the bulky neopentyl group in **3** disfavors an outer-sphere pathway, we note that the inner-sphere pathway at the less sterically encumbered [(**L1**)Ni(I)Me] was still found to be favored computationally by 6.6 kcal mol^–1^ (Figure S39). This finding is important, as it supports the notion that the ancillary ligand can influence the mechanism of CO_2_ insertion.^24,26^

**Figure 2:**
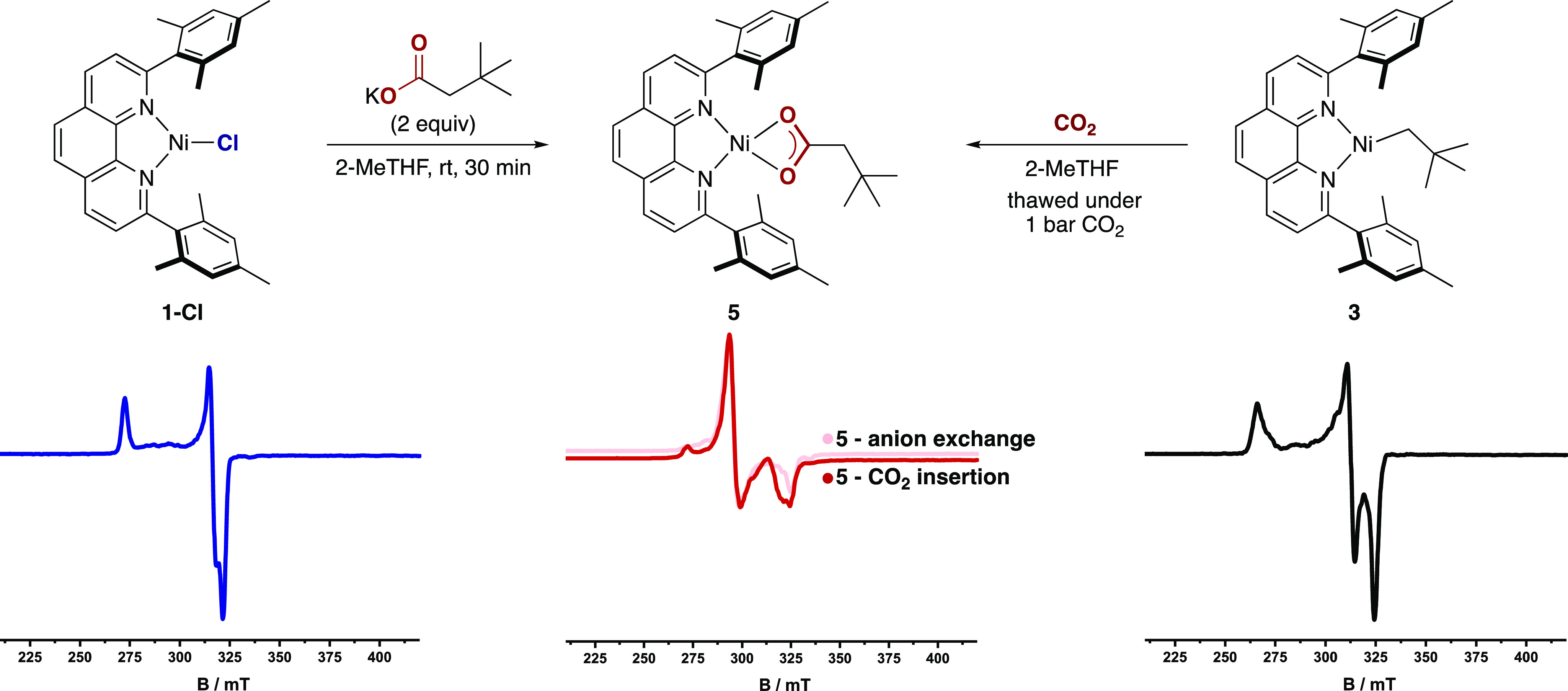
CO_2_ insertion at Ni(I). (top) Anion metathesis reaction (left) and CO_2_ insertion into **3** (right). (bottom) Changes in the 77 K X-band EPR spectra of **1-Cl** (left, *g*_*x*_ = 2.084, *g*_*y*_ = 2.119, *g*_*z*_ = 2.461) after anion metathesis and after CO_2_ insertion at **3** (right, *g*_*x*_ = 2.065, *g*_*y*_ = 2.145, *g*_*z*_ = 2.519) to form **5** (center, *g*_*x*_ = 2.299, *g*_*y*_ = 2.272, *g*_*z*_ = 2.064).

**Figure 3:**
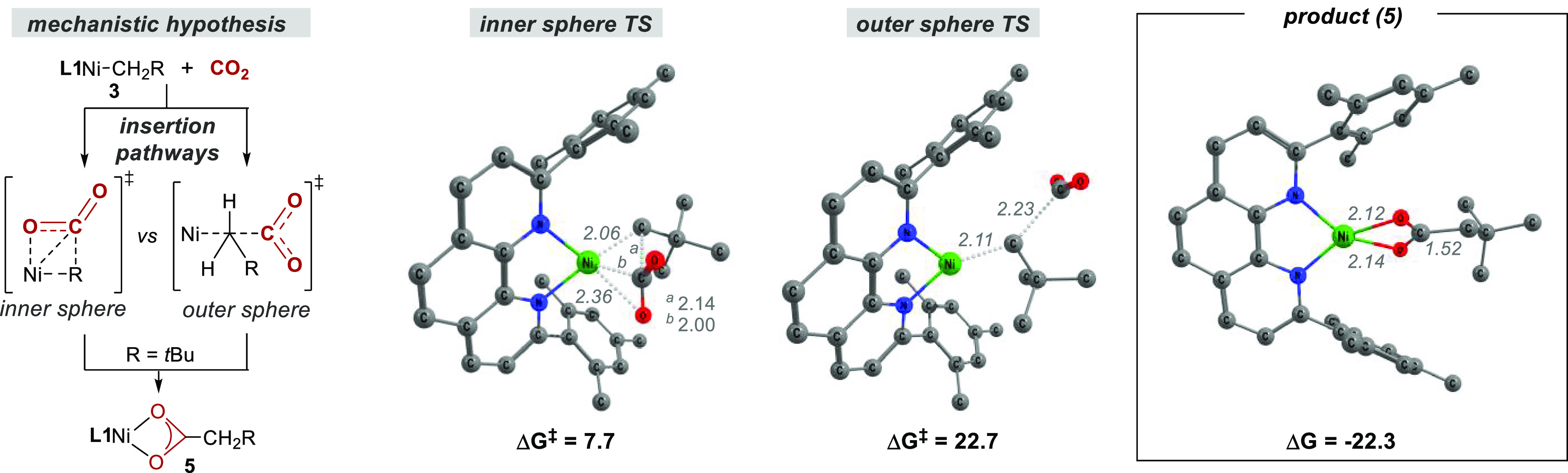
Optimized TS geometries for inner-sphere vs outer-sphere CO_2_ insertion and the optimized geometry of **5** (PBE-D3BJ/def2-TZVP/IEFPCM, H atoms omitted, distances in Å, energies in kcal mol^–1^ relative to **3** + free CO_2_, 298.15 K).

**Scheme 3:**
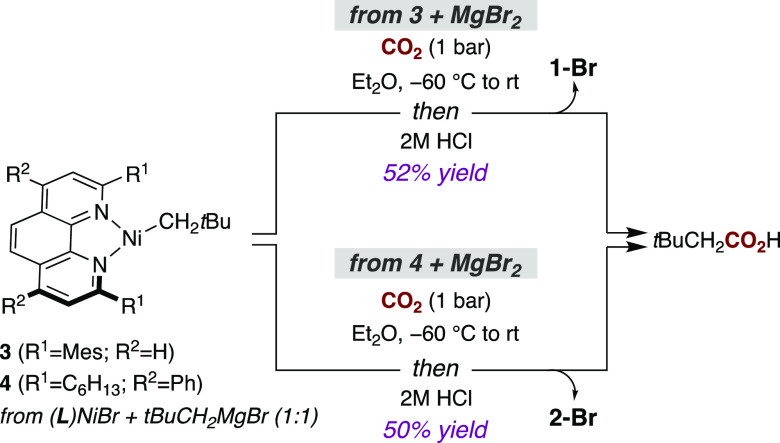
CO_2_ Insertion En Route to *t*BuCH_2_CO_2_H

Given the relevance of **L2** in Ni-catalyzed carboxylation reactions, CO_2_ insertion at **4** was also studied. Although the sensitivity of **4** prevented workup to remove MgBr_2_ (the byproduct obtained by reacting (**L2**)Ni(I)Br with 1 equiv of neopentylMgBr), a 50% yield of *tert*-butylacetic acid was obtained upon exposure of a cold solution of **4** to CO_2_ (1 bar) and then to dilute HCl (Scheme 3, bottom). Interestingly, this reaction mixture rapidly turned blue upon CO_2_ addition, and only **2-Br** was observed by EPR spectroscopy. This suggested that the **L2** carboxylate complex [(**L2**)Ni(I)O_2_CCH_2_*t*Bu] (**6**) resulting from CO_2_ insertion at Ni(I) underwent halide exchange with MgBr_2_ to form blue **2-Br**. This was confirmed by the addition of MgBr_2_ to salt-free **5**. Given the wide number of Ni-catalyzed reductive coupling reactions that employ MgX_2_ (X = Br, Cl) additives,^27^ the formation of **2-Br** from in situ-generated **6** provides support for the formation of Ni(I) halide complexes prior to reduction to the propagating Ni(0)**L**_*n*_ species.^28^

In conclusion, we have investigated the synthesis and CO_2_ insertion reactivity of Ni(I)–alkyl complexes bearing catalytically relevant phen ligands. We have obtained experimental evidence for the rapid insertion of CO_2_ into Ni(I)–C bonds, a long-presumed elementary step in the reductive carboxylation of alkyl (pseudo)halides. Given the widespread use of phen ligands in Ni-catalyzed reactions, these results are expected to guide new investigations into the catalytic relevance of Ni(I)–alkyl complexes. Further investigations along these lines are currently underway in our laboratories.

## Supporting Information Available

Crystallographic data for **1-Cl** (CIF)
Crystallographic data for **2-Cl** (CIF)
Crystallographic data for **3** (CIF)
Cartesian coordinates of calculated structures (XYZ)
Experimental procedures, spectra, crystallographic details, and computational details (PDF)

## Supplementary Material

ja0c04695_si_001.cif

ja0c04695_si_002.cif

ja0c04695_si_003.cif

ja0c04695_si_004.xyz
