# Supplementary material for: Ni(I)–Alkyl Complexes Bearing Phenanthroline Ligands: Experimental Evidence for CO2 Insertion at Ni(I) Centers
Source: J Am Chem Soc. 2020 Jun 10;142(25):10936–41. doi: 10.1021/jacs.0c04695 (PMC7351122; doi:10.1021/jacs.0c04695)
Supplement: Supplementary file 5 — ja0c04695_si_005.pdf [file ja0c04695_si_005.pdf]

Supporting Information

Ni(I)–Alkyl Complexes Bearing Phenanthroline Ligands: Experimental Evidence for CO_2 Insertion at Ni(I) Centers

Rosie J. Somerville,[†¶] Carlota Odena,[†¶‡] Marc F. Obst,[¥‡] Nilay Hazari,[£*] Kathrin H. Hopmann[¥*] and Ruben Martin[†§*]

[†] Institute of Chemical Research of Catalonia (ICIQ), The Barcelona Institute of Science and Technology, Av. Països Catalans 16, 43007 Tarragona, Spain
[¶] Departament de Química Analítica i Química Orgànica, Universitat Rovira i Virgili, c/Marcel·lí Domingo, 1, 43007 Tarragona, Spain
[§] ICREA, Passeig Lluís Companys, 23, 08010 Barcelona, Spain
[¥] Hylleraas Center for Quantum Molecular Sciences, Department of Chemistry, UiT The Arctic University of Norway, N-9307 Tromsø, Norway

[£] Department of Chemistry, Yale University, P.O. Box 208107, New Haven, Connecticut 06520, United States

Corresponding authors

Ruben Martin: rmartinromo@iciq.es
Kathrin Hopmann: kathrin.hopmann@uit.no

Table of Contents

General considerations **2**

Preparation of nickel complexes **3**

Nickel(II) dihalide precursors 3

Nickel(I) halide complexes 5

Nickel(I) alkyl complexes 9

In situ synthesis of Nickel(I)-alkyl complexes **10**

Unsuccessful alkylation reactions of 1-Cl and 1-Br 12

Nickel(I) carboxylate complex **15**

CO_2 insertion into Nickel(I)–alkyl complexes **15**

Catalytic carboxylation reactions **17**

IR spectra **19**

NMR spectra of halide complexes **22**

NMR spectra of alkyl complexes **26**

NMR spectra of 5 .... **27**

Crystallographic data **28**

Computational Details **31**

References **34**

[‡]These authors contributed equally.

General considerations

Reagents. Ni(COD)$_2$ and NiI$_2$ (99.99% trace metal basis) were purchased from Strem Chemicals. Neocuproine, bathocuproine, Mn powder (99.99% trace metal basis), 1-bromoheptane, and 2-bromoheptane were purchased from Sigma Aldrich. 2,9-Dichlorophenanthroline was purchased from Fluorochem. Anhydrous *N,N*-dimethylformamide (DMF) was purchased from Acros Organics. **L3** (*t*Bu-Xantphos) was purchased from DSC.
Neopentylmagnesium bromide was synthesized following a reported procedure.[1]
L1[2] and **L2**[3,4] were synthesised according to literature procedures. Potassium carboxylate salt *t*BuCH$_2$CO$_2$K was synthesised by combining 3,3-dimethylbutyric acid with KOH in methanol followed by removal of the volatiles. All other reagents were purchased from commercial sources and used without further purification.

Solvents. Hydrocarbon solvents, 2-MeTHF, toluene-d$_8$, THF-d$_8$ and benzene-d$_6$ were degassed by the appropriate method (sparging or three freeze-pump-thaw cycles) then dried over 4Å molecular sieves and stored in the glovebox. Inhibitor-free THF and Et$_2$O were purified with an Innovative Technologies solvent purification system. CDCl$_3$ was used as received for NMR of air-stable Ni(II) complexes.

Analytical methods. Flash chromatography was performed with Sigma Aldrich technical grade silica gel 60 (230-400 mesh). Thin layer chromatography was carried out using Merck TLC Silica gel 60 F254. NMR spectra were recorded on Bruker Avance Ultrashield 300, 400, or 500 MHz spectrometers, with chemical shifts reported in parts per million (ppm) and coupling constants, *J*, reported in hertz. IR spectra were obtained with a Bruker FT-IR Alpha spectrometer inside the glovebox.

Continuous wave (CW) X-band EPR spectra were obtained using a Bruker EMX Micro X-band spectrometer using a Bruker ER 1164 HS resonator. Spectra were simulated using SpinFit within Xenon. The samples were cooled to 77 K in a Suprasil finger dewar (Wilmad-LabGlass) filled with liquid nitrogen. The spectral data were collected with the following spectrometer settings: microwave power = 0.56 mW; centre field = 3250 G, sweep width = 2500 G, sweep time = 35.07 s, modulation frequency = 100 KHz, modulation amplitude = 10 G, power attenuation = 25 dB, time constant = 20.48 ms. Sweep width of 3500 G and power of 0.346 mW were employed for obtaining the yield for the in situ syntheses of **3** and **4**. Simulations, g values, and frequencies are provided alongside the characterisation data of the complexes.
Evans method[5,6] solution magnetic susceptibility measurements were performed for [NiBr$_2$(**L2**)], **1-Cl**, **1-Br**, and **3**.

Dr Brandon Q. Mercado (Yale University) collected the X-ray data for **1-Cl** and refined and solved the structure (see X-ray crystallography section).

Preparation of nickel complexes

Nickel(II) dihalide precursors

[$NiCl_2$(L1)]

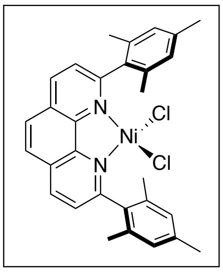

In a 12 mL vial, **L1** (175.4 mg, 0.421 mmol, 1 equiv) and $NiCl_2$·DME (92.5 mg, 0.421 mmol, 1 equiv) were combined in 4 mL THF. Stirring at room temperature for 45 minutes followed by sonication for 10 minutes gave a purple suspension. This was combined with 4 mL Et_2O then filtered, washed with further Et_2O (2 x 5 mL), and dried under vacuum to give paramagnetic [$NiCl_2$(**L1**)] as a pink solid (197 mg, 86% yield).

^{1}H NMR (500 MHz, $CDCl_3$): *δ* 73.72 (s, 2H, **L1**), 25.28 (s, 2H, **L1**), 24.71 (s, 2H, **L1**) 9.63 (s, 4H, **L1**-mes), 5.30 (s, 12 H, *ortho*-CH_3), 4.96 (br, $\Delta\nu_{1/2}$ = 41 Hz, 6 H, *para*-CH_3).
EA Calcd. C, 65.98; H, 5.17; N, 5.13; Found: C, 65.26; H, 5.40; N, 4.88.

[$NiBr_2$(L1)]

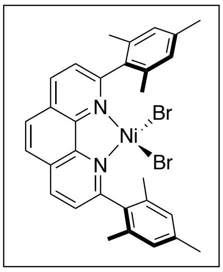

In a 12 mL vial, **L1** (80 mg, 0.192 mmol, 1 equiv) and $NiBr_2$·DME (59 mg, 0.192 mmol, 1 equiv) were combined in 2 mL THF. Sonication for 10 minutes gave a purple suspension that was filtered, washed with Et_2O, and dried under vacuum to give paramagnetic [$NiBr_2$(**L1**)] as a dark pink solid (101 mg, 83%).

^{1}H NMR (500 MHz, $CDCl_3$): *δ* 71.55 (s, $\Delta\nu_{1/2}$ = 60 Hz, 2H, **L1**), 25.33 (s, 2H, **L1**), 24.27 (s, 2H, **L1**), 8.76 (s, 4H, **L1**-mes), 4.94 (s, 12 H, *ortho*-CH_3), 4.33 (br, $\Delta\nu_{1/2}$ = 70 Hz, 6 H, *para*-CH_3).
EA Calcd. C, 56.74; H, 4.44; N, 4.41; Found: C, 52.31; H, 4.41; N, 4.05. ($CHCl_3$ contaminant likely)

[NiCl$_2$(L2)]

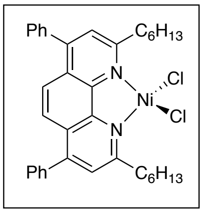

In a 12 mL vial, **L2** (200.6 mg, 0.401 mmol, 1 equiv) and $NiCl_2$·DME (88.0 mg, 0.401 mmol, 1 equiv) were combined in 3 mL THF. Stirring at room temperature for 45 minutes followed by sonication for 10 minutes gave a purple suspension. This was combined with 10 mL pentane then filtered, washed with further Et_2O (3 x 5 mL), and dried under vacuum to give paramagnetic [NiCl$_2$(**L2**)] as a pink solid (234.8 mg, 93% yield).

^{1}H NMR (400 MHz, $CDCl_3$): δ 82.40 (s, 2H, **L2**), 30.26 (br, $\Delta\nu_{1/2}$ = 450 Hz, 4H, CH_2), 28.40 (s, 2H, **L2**), 17.89 (s, 4H) , 9.44 (m, 4H, **L2**-Ph), 8.76 (m, 2H, **L2**-Ph), 7.23 (m, 4H, **L2**-Ph), 7.08 (s, 4H, CH_2), 3.95(s, 4H, CH_2), 2.73 (s, 4H, CH_2), 1.58 (m, 6H, CH_3).
EA Calcd. C, 68.60; H, 6.40; N, 4.44; found: C, 68.09; H, 6.27; N, 4.50.

[NiBr$_2$(L2)]

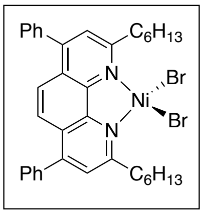

Synthesised following a modified literature procedure.[7]

To a mixture of $NiBr_2 \cdot 3H_2O$ (99.0 mg, 0.363 mmol, 1 equiv) and **L2** (181.8 mg, 0.363 mmol, 1 equiv) was added 10 mL ethanol. The mixture was stirred under air at 40 °C for 30 minutes, then the purple solid was filtered off and washed with 2 x 10 mL anhydrous ethanol followed by 2 x 10 mL Et_2O. Drying under vacuum gave paramagnetic [NiBr$_2$(**L2**)] as a pink solid (166.4 mg, 64% yield).

^{1}H NMR (400 MHz, $CDCl_3$): δ 80.56 (br, $\Delta\nu_{1/2}$ = 80 Hz, 2H, **L2**), 29.16 (s, 2H, **L2**), 18.35 (br, $\Delta\nu_{1/2}$ = 800 Hz, 4H, CH_2), 13.98 (br, $\Delta\nu_{1/2}$ = 110 Hz, 4H, CH_2), 9.31 (m, 4H, **L2**-Ph), 8.71 (m, 2H, **L2**-Ph), 7.17 (m, 4H, **L2**-Ph), 4.76 (s, 4H, CH_2), 2.84 (s, 4H, CH_2), 2.19 (s, 4H, CH_2), 1.34 (s, 6H, CH_3).
Magnetic susceptibility (Evans method) $\boldsymbol{\mu_{eff}}$ = 2.88 μ_B
EA Calcd. C, 60.12; H, 5.61; N, 3.89; Found C: 59.95; H: 5.47; N: 3.99

Nickel(I) halide complexes

The four Ni(I)-halide complexes below were synthesised by comproportionation (Scheme S1)

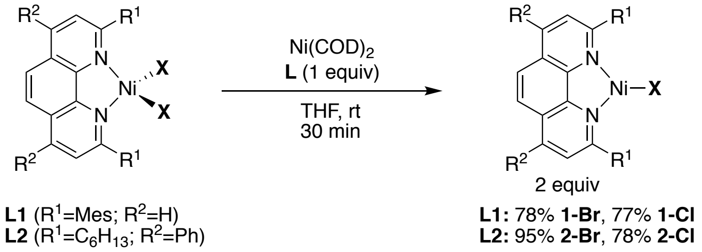

Scheme S1. General method for synthesis of nickel(I) halide complexes.

[(L1)NiCl] (1-Cl)

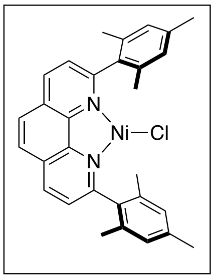

L1 (42.5 mg, 0.102 mmol, 1 equiv), [$NiCl_2$(**L1**)] (55.7 mg, 0.102 mmol, 1 equiv), and $Ni(COD)_2$ (28.1 mg, 0.102 mmol, 1 equiv) were combined in 2 mL THF. After stirring for 10 min at room temperature, the solution was filtered through glass fibre filter paper. This was washed with an additional 2.5 mL THF. The volatiles were then removed, the solid washed with pentane, then dried under vacuum to give **1-Cl** as a dark blue powder (80 mg, 77%).

^{1}H NMR (500 MHz, toluene-d_8): δ 33.38 (br, $\Delta\nu_{1/2}$ = 1200 Hz (approx.), 2H, **L1**), 10.75 (br, $\Delta\nu_{1/2}$ = 300 Hz (approx.), 2H, **L1**), 6.14 (br, $\Delta\nu_{1/2}$ = 850 Hz), 5.03 (br, $\Delta\nu_{1/2}$ = 400 Hz (approx.)), 2.11 (s).
The ^{1}H NMR spectrum is very broad and the signals were not able to be assigned to particular proton environments.
Magnetic susceptibility (Evans method) μ_{eff} = 1.94 μ_B
EPR spectrum simulated with g_x = 2.084, g_y = 2.119, g_z = 2.461. lineshape = 0.8. The continuous wave EPR spectrum was obtained in 2-MeTHF at 9.386 GHz.

Single crystals of **1-Cl** were grown from toluene/pentane at −35 °C.

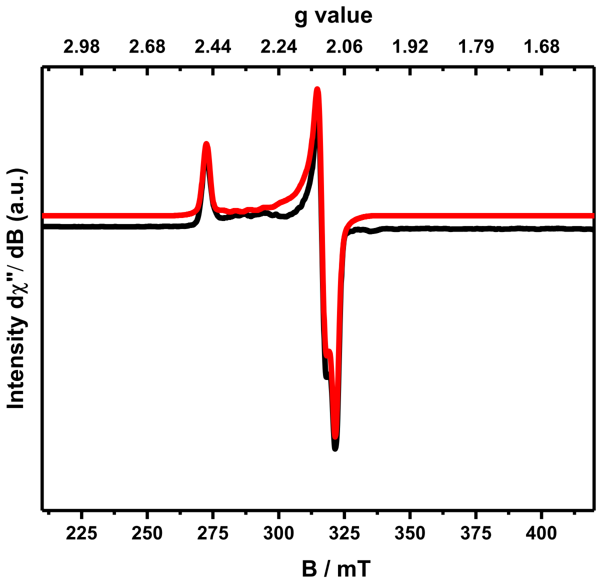

Figure S1. EPR spectrum of 1-Cl (77 K, 2-MeTHF). red = simulated spectrum.

[(L1)NiBr] (1-Br)

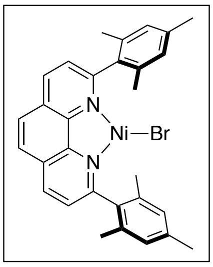

L1 (52.7 mg, 0.127 mmol, 1 equiv), [$NiBr_2$(**L1**)] (80.4 mg, 0.127 mmol, 1 equiv), and $Ni(COD)_2$ (28.1 mg, 0.127 mmol, 1 equiv) were combined in 4 mL THF. After stirring for 15 min at room temperature, the solution was filtered through glass fibre filter paper. This was washed with an additional 1 mL THF. The volatiles were then removed, the solid washed with pentane, then dried under vacuum to give **1-Br** as a dark blue powder (109.7 mg, 78%).

^{1}H NMR (500 MHz, toluene-d_8): δ 32.72 (br, $\Delta\nu_{1/2}$ = 1400 Hz (approx.), **L1**), 11.18 (br, $\Delta\nu_{1/2}$ = 255 Hz, **L1**), 5.94 (br s), 5.52 (br, $\Delta\nu_{1/2}$ = 360 Hz), 2.48 (br s).

The ^{1}H NMR spectrum is very broad and the signals were not able to be assigned to particular proton environments.

Magnetic susceptibility (Evans method) μ_{eff} = 1.87 μ_B

EPR spectrum simulated with g_x = 2.093, g_y = 2.129, g_z = 2.469. lineshape = 1. The continuous wave EPR spectrum was obtained at 9.389 GHz.

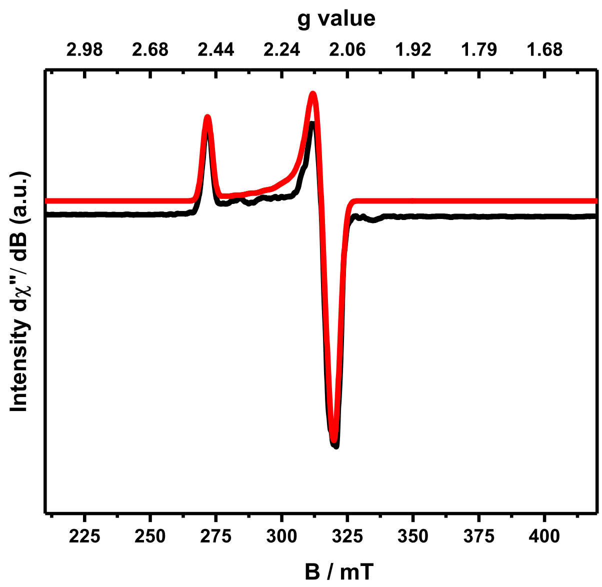

Figure S2. EPR spectrum of 1-Br (77 K, 2-MeTHF). red = simulated spectrum.

[(L2)NiCl] (2-Cl)

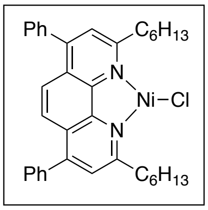

L2 (35.4 mg, 0.0707 mmol, 1 equiv), [$NiCl_2$(**L2**)] (44.6 mg, 0. 0707 mmol, 1 equiv), and $Ni(COD)_2$ (19.5 mg, 0. 0707 mmol, 1 equiv) were combined in 1 mL THF. After stirring for 1 h at room temperature, the solution was filtered through glass fibre filter paper. This was washed with an additional 1 mL THF. Pentane (2 mL) was then added and the solution placed in the freezer (−35 °C) overnight. The solid was filtered off, washed with cold pentane (2 mL), then dried under vacuum to give **2-Cl** as a deep blue crystalline solid (66.2 mg, 78%).

Crystals suitable for X-ray diffraction were obtained from this THF/pentane mixture at −35 ºC.

^{1}H NMR (400 MHz, toluene-d_8): δ 37.07 (br, $\Delta\nu_{1/2}$ = 1500 Hz (approx.), 2H, **L2**), 12.61 (br, $\Delta\nu_{1/2}$ = 300 Hz, 2H), 8.74 (s, **L2**-Ph), 8.62 (s, **L2**-Ph) [δ(8.74+8.62) = 10H], 5.73 (s, 6H, hexyl signals), 1.29 (br m, 20 H, hexyl signals). **EPR** spectrum simulated with g_x = 2.089, g_y = 2.114, g_z = 2.449. lineshape = 0.9. The continuous wave EPR spectrum was obtained at 9.384 GHz.

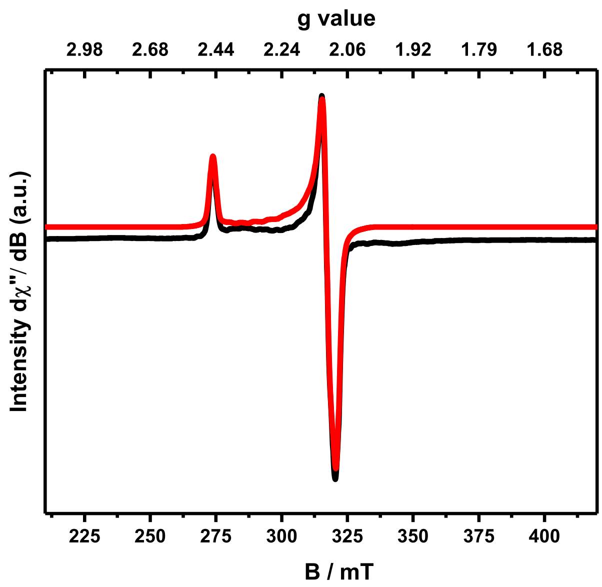

Figure S3. EPR spectrum of 2-Cl (77 K, 2-MeTHF). red = simulated spectrum.

[(L2)NiBr] (2-Br)

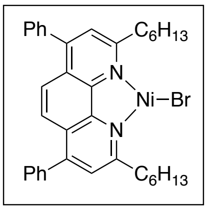

L2 (91.8 mg, 0.183 mmol, 1 equiv), [$NiBr_2$(**L2**)] (131.9 mg, 0.183 mmol, 1 equiv), and $Ni(COD)_2$ (50.44 mg, 0.183 mmol, 1 equiv) were combined in 3 mL THF. After stirring for 40 min at room temperature, the solution was filtered through glass fibre filter paper. This was washed with an additional 2 mL THF. The volatiles were then removed, the solid washed with pentane, then dried under vacuum to give **2-Br** as a dark blue powder (224 mg, 95%).

^{1}H NMR (400 MHz, toluene-d_8): δ 35.81 (very broad s, $\Delta\nu_{1/2}$ = 1500 Hz (approx.)), 12.41 (br, $\Delta\nu_{1/2}$ = 215 Hz, 2H, **L2**), 8.65 (s, **L2**-Ph), 8.51 (s, $\Delta\nu_{1/2}$ = 1500 Hz, **L2**-Ph) [δ(8.74+8.62) = 8H], 5.80 (s, 5H), 1.26 (m). Some signals are extremely broad.
EPR spectrum simulated with g_x = 2.090, g_y = 2.126, g_z = 2.458. lineshape = 1. The continuous wave EPR spectrum was obtained at 9.390 GHz.

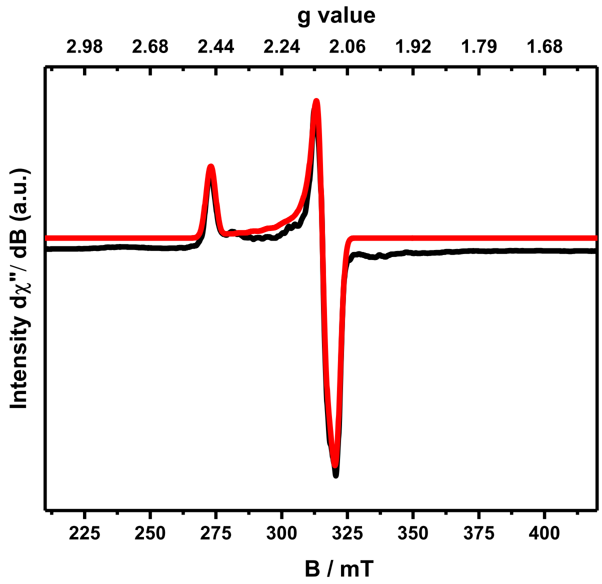

Figure S4. EPR spectrum of 2-Br (77 K, 2-MeTHF). red = simulated spectrum.

Nickel(I) alkyl complexes

[(L1)Ni(CH_2*t*Bu)] (3)

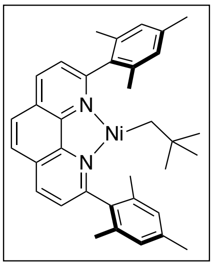

A suspension of **1-Br** (7.8 mg, 0.014 mmol, 1 equiv) in 1.5 mL Et_2O was cooled to −35 ºC. A solution of neopentylMgBr (140 µL, 0.2 M in THF, 2 equiv) was then added. The vial was agitated until the dark blue **1-Br** had disappeared (ca. 5 min). The deep green reaction mixture was filtered through glass fibre filter paper to remove unreacted **1-Br** and some $MgBr_2$, then dioxane (200 µL) was added to precipitate the remaining magnesium salts. The cloudy solution was placed in the glovebox freezer for 10 minutes (−35 ºC) then filtered through 1.5 cm of celite in a pipette. Volatiles were removed then the dark green residue was extracted with 3 mL pentane. The pentane extracts were filtered through celite, then pentane was removed to give **3** as a dark green solid (3.9 mg, 50%).

Single crystals were obtained from an Et_2O/pentane mixture at −35 ºC.

^{1}H NMR (500 MHz, toluene-d_8): δ 55.35 (br, $\Delta\nu_{1/2}$ = 1500 Hz (approx.), **L1**), 43.97 (br, $\Delta\nu_{1/2}$ = 1700 Hz (approx.), **L1**), 13.68 (br, $\Delta\nu_{1/2}$ = 185 Hz), 10.74 (br, $\Delta\nu_{1/2}$ = 2000 Hz (approx.)), 6.82 (br, $\Delta\nu_{1/2}$ = 650 Hz (approx.)), 2.06 (br, $\Delta\nu_{1/2}$ = 250 Hz (approx.)).
The broad and overlapping signals between 0–10 ppm precluded assignment.
Magnetic susceptibility (Evans method) μ_{eff} = 2.23 μ_B
EPR spectrum simulated with g_x = 2.065, g_y = 2.145, g_z = 2.519. lineshape = 0.95. The continuous wave X-band EPR spectrum was obtained at 9.390 GHz.

A small amount of **1-Br** is visible as a shoulder on the right-hand side of g_z in the black **3** spectrum (measured).

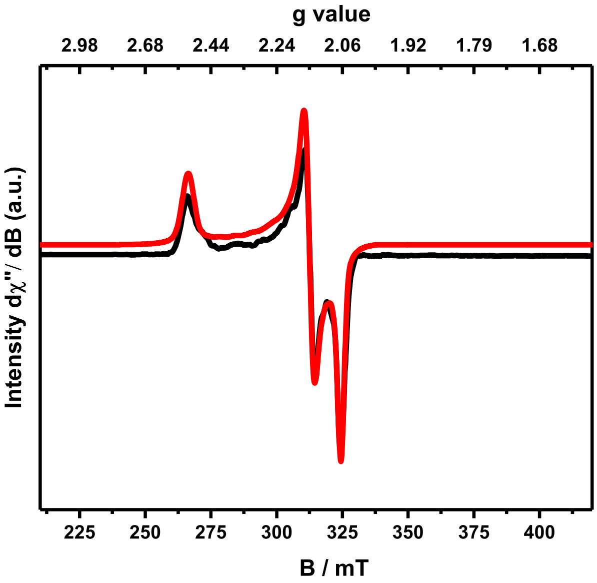

Figure S5. EPR spectrum of isolated 3 (77 K, 2-MeTHF). red = simulated spectrum.

Attempts to isolate **4** in the same manner as above but keeping all glassware, solvents, and celite in the glovebox cold well failed – the initially deep emerald green solution became browner during manipulations and EPR spectra showed significant loss of intensity of the Ni(I) signal and the appearance of organic radical at g ca. 2.0.

In situ synthesis of Nickel(I)-alkyl complexes

[(L1)Ni(CH₂*t*Bu)] (3)

1-Br (5.0 mg, 0.0098 mmol, 1 equiv) was suspended in 1 mL 2-MeTHF. A solution of neopentylMgBr (145 µL, 0.029 mmol, 3 equiv) was then added and the vial agitated briefly to ensure reaction of the **1-Br**. The resulting deep green **3** solution was transferred to an EPR tube.

EPR spectrum simulated with $g_x = 2.065$, $g_y = 2.145$, $g_z = 2.519$. lineshape = 0.95. The continuous wave X-band EPR spectrum was obtained at 9.385 GHz.

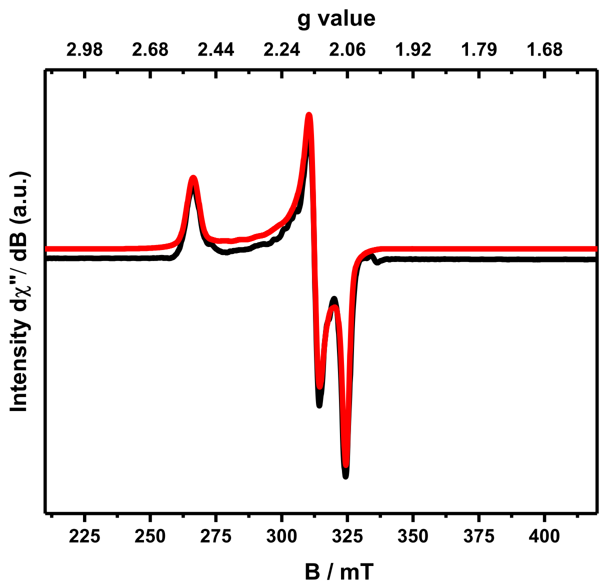

Figure S6. EPR spectrum of in situ generated 3 (77 K, 2-MeTHF).

The reaction was repeated and integration of the resulting EPR spectrum against solutions of known Cu(II) concentration gave a 68% yield of **3**:

1-Br (3.5 mg, 0.0055 mmol, 1 equiv) was suspended in 1 mL 2-MeTHF and cooled for 10 minutes in the freezer (−35 ºC). A solution of neopentylMgBr (35.75 µL, 0.0072 mmol, 1.3 equiv) was then added and the vial agitated for 5 minutes at room temperature to ensure reaction of the **1-Br**. The resulting deep green **3** solution was transferred to an EPR tube.

[(L2)Ni(CH$_2$*t*Bu)] (4)

2-Br (3.5 mg, 0.0055 mmol, 1 equiv) was suspended in 1 mL 2-MeTHF and cooled for 30 minutes in cold well of the glove box (< −100 ºC). A solution of neopentylMgBr (81 µL, 0.016 mmol, 3 equiv) was then added, the vial agitated briefly, then the deep green solution was transferred to a chilled EPR tube placed inside an aluminium block that had been kept in the cold well. This was removed from the glovebox then the tube frozen in liquid nitrogen.

EPR spectrum in Figure S7 simulated with $g_x = 2.074$, $g_y = 2.138$, $g_z = 2.506$. lineshape = 1. The continuous wave X-band EPR spectrum was obtained at 9.385 GHz.

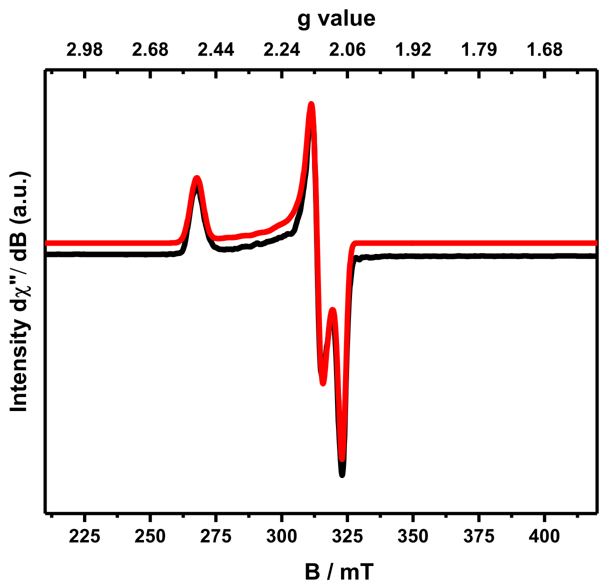

Figure S7. EPR spectrum of in situ generated 4 (77 K, 2-MeTHF). red = simulated spectrum.

The reaction was repeated and integration of the resulting EPR spectrum against solutions of known Cu(II) concentration gave a 57% yield of **4**:

2-Br (3.0 mg, 0.0055 mmol, 1 equiv) was suspended in 1 mL 2-MeTHF and cooled for 30 minutes in cold well of the glove box (< −100 ºC). A solution of neopentylMgBr (82.5 µL, 0.017 mmol, 3 equiv) was then added, the vial agitated briefly, then the deep green solution was transferred to a chilled EPR tube placed inside an aluminium block that had been kept in the cold well. This was removed from the glovebox then the tube frozen in liquid nitrogen.

Unsuccessful alkylation reactions of 1-Cl and 1-Br

Reactions between **L1** halide complexes **1-Cl** and **1-Br** and MeMgCl or EtMgBr were carried out as for the **2-Br** alkylation reactions described above. The resulting EPR spectra (77 K, 2-MeTHF glass) are shown below.

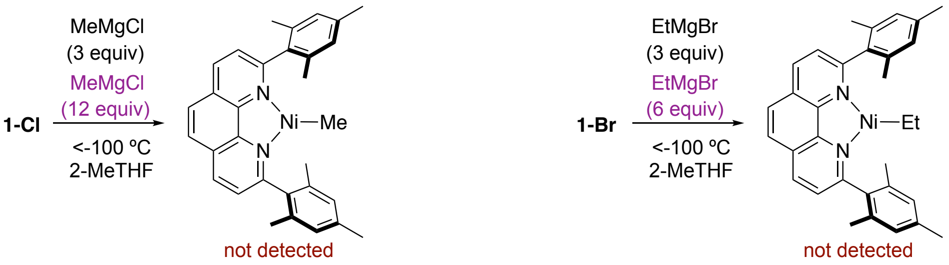

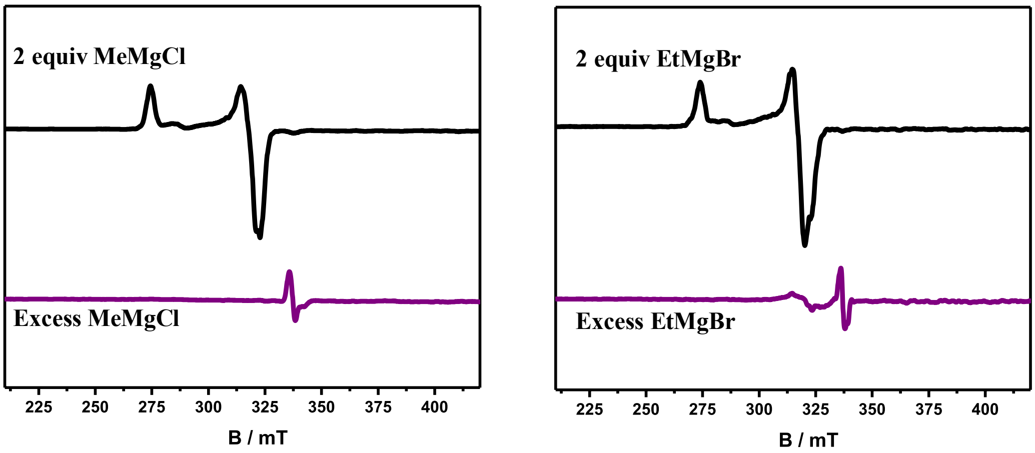

Figure S8. EPR spectra (77 K, 2-MeTHF) of reactions between [(L1NiX] and MeMgCl (left) and EtMgBr (right).

When the reaction of **1-Cl** with 2 equiv MeMgCl was carried out in toluene-d$_8$ and analysed by ^{1}H NMR spectroscopy, no clear paramagnetic signals could be identified. However, block-shaped green crystals were obtained. The crystals diffracted very poorly but the connectivity structure showed a [Ni(**L1**)]$_4$ tetramer (Figure S9). The Ni(**L1**) units coordinated to each C5–C6 bond of **L1** of another Ni(**L1**) unit.

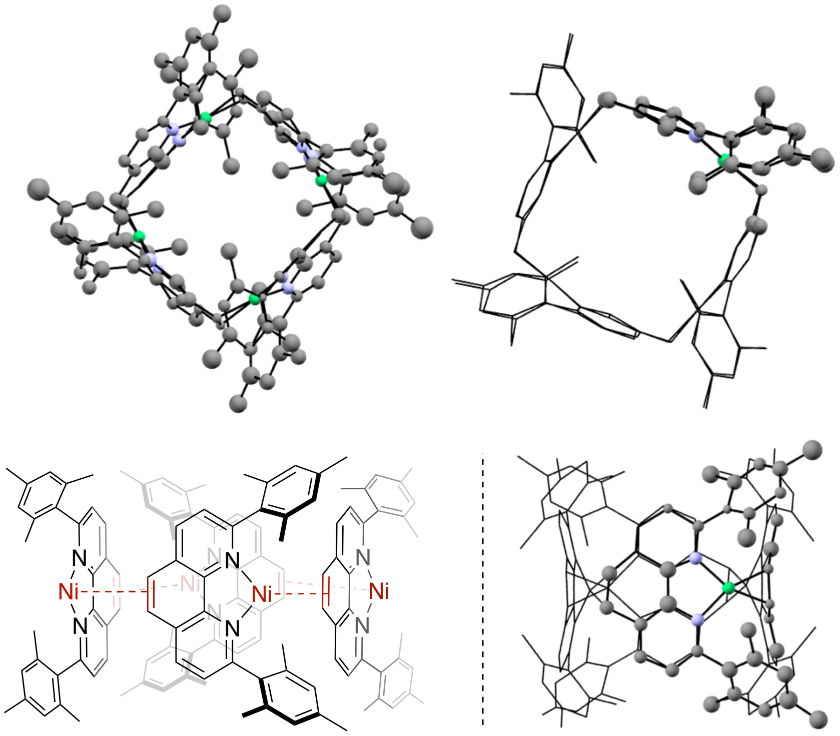

Figure S9. Connectivity structure of [Ni(L1)]$_4$ tetramer (two molecules in the asymmetric unit).

When **1-Br** was reacted with EtMgBr in THF-d$_8$, green crystals formed inside the NMR tube. A [Ni(**L1**)]$_3$ trimer structure was identified (Figure S10). Again, the crystals did not diffract well so we can only provide connectivity information.

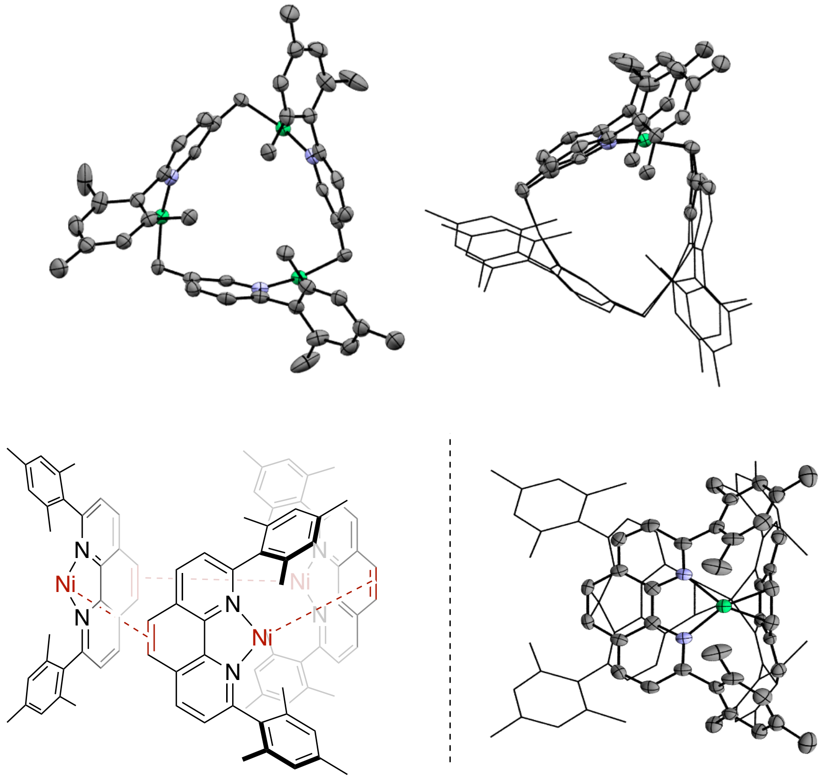

Figure S10. [Ni(L1)]$_3$. Hydrogen atoms and two THF molecules of crystallisation not shown.

There are no examples in the Cambridge Structural Database (as of March 2020) of complexes where the metal coordinates to the C5–C6 position of a phenanthroline ligand. The angle between (**L1**)Ni and the **L1** backbone to which it is coordinated is approximately 105º, consistent with reported complexes where a L_nNi(0) group is coordinated to a π system.[8–12] The concavity of the **L1** backbone in both structures is unusual, however, and suggests that the phenanthroline ligand has lost aromaticity and is acting as two pyridine ligands separated by a π-bond spacer (where Ni(**L1**) coordinates) (Figure S11). The steric bulk of **L1** likely prevents formation of Ni(**L1**)$_2$, so this unusual interaction may be stabilising Ni(0) formed upon decomposition or reduction.

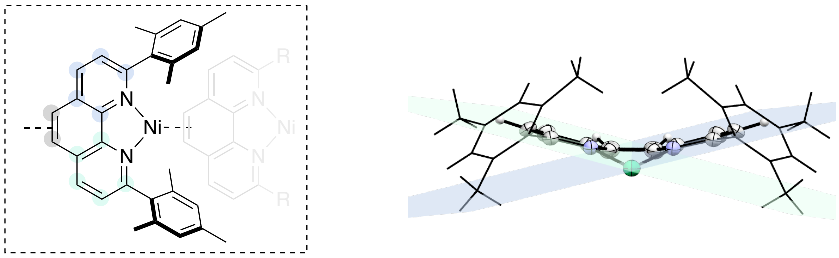

Figure S11. Fragment of [Ni(L1)]$_3$ to illustrate angle between planes (ca. 23°).

Nickel(I) carboxylate complex

Generation of 5 via anion metathesis

2-MeTHF:

1-Cl (6.2 mg, 0.012 mmol, 1 equiv) and KO_2CCH_2*t*Bu (3.74 mg, 0.024 mmol, 2 equiv) were suspended in 2-MeTHF (1 mL) and stirred at room temperature for 30 minutes. After this time, a 0.4 mL aliquot was transferred to an EPR tube. The tube was sealed, taken out of the glovebox and the sample frozen in liquid nitrogen for analysis by EPR spectroscopy (77 K). NOTE: When the same reaction was repeated with 3 equiv KO_2CCH_2*t*Bu (45 min) or with 1.5 equiv KO_2CCH_2*t*Bu for 90 min, the resulting EPR signal for **5** was lower in intensity than that of the 2 equiv/30 min reaction.

Toluene:

1-Cl (6.2 mg, 0.012 mmol, 1 equiv) and KO_2CCH_2*t*Bu (2.4 mg, 0.0156 mmol, 1.3 equiv) were suspended in toluene (1 mL) and stirred at room temperature for 5 minutes. After this time, a 0.4 mL aliquot was transferred to an EPR tube. The tube was sealed, taken out of the glovebox and the sample frozen in liquid nitrogen for analysis by EPR spectroscopy (77 K). One reaction was carried out in toluene-d_8 (0.7 mL) and after stirring for 5 minutes at room temperature, the solution was filtered through glass fibre filter paper and transferred to a J Young NMR tube in order to record the ^1H NMR spectrum.

For reactions where the IR spectrum was to be measured, only 1 equiv of KO_2CCH_2*t*Bu was employed.

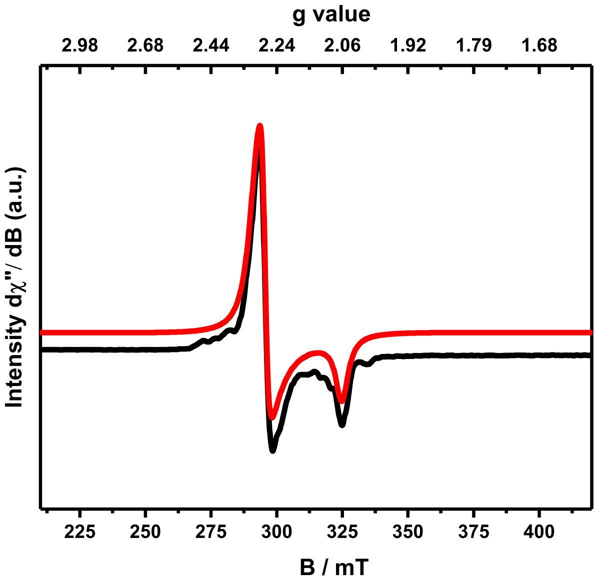

Figure S12. EPR spectrum of carboxylate complex 5 synthesised by anion metathesis. Simulated spectrum shown in red with: $g_x = 2.299$, $g_y = 2.272$, $g_z = 2.064$ freq: 9.388 GHz. lineshape = 0.

CO_2 insertion into Nickel(I)–alkyl complexes

Neopentyl complexes were either generated in situ with 1 equiv neopentylMgBr or isolated **3** was employed. An EPR spectrum of the neopentyl complex was always collected prior to CO_2 addition. Reactions involving **L2** neopentyl complex **4** were maintained at low temperature by working in the glovebox cold well and transferring the EPR tube from the glovebox while it was inside a cold aluminium block.

Procedure for CO_2 addition – small scale

The desired alkyl complex was dissolved in 2-MeTHF or prepared from the appropriate [(**L**)NiBr] complex and neopentylMgBr inside the glovebox. The solution was then transferred to a resealable EPR tube and frozen in liquid N_2. The atmosphere in the tube was carefully exchanged for 0.1 bar CO_2, then the reaction mixture was thawed to ca. −60 ºC (ethanol dry ice bath, thermometer), briefly agitated, and finally refrozen under vacuum.

Addition of $^{13}CO_2$ was carried out in a similar manner using a 1 L lecture bottle (Sigma Aldrich). Pressure inside the (new) bottle was 1.36 atm.

Procedure for CO_2 addition – quenching experiments

For larger scale experiments (Scheme 3 in manuscript), a Teflon-sealed Schlenk ampoule containing an Et_2O solution of the appropriate [(**L**)NiBr] complex (generated in situ from 1:1 **1-Br** or **2-Br** and neopentylMgBr at room temperature and in the cold well of the glovebox, respectively) was frozen, placed under vacuum, then warmed slightly prior to CO_2 addition to avoid condensing CO_2 (e.g. thawed from frozen in a –60 °C cold bath for the reaction shown below). The solution was then warmed to room temperature and agitated for 15 minutes. Afterwards, the reaction was quenched with 2M HCl and the Et_2O layer was extracted. A ^1H NMR spectrum was recorded using TMS or 1,3,5-trimethoxybenzene as internal standard.

2-Br (43 mg, 0.067 mmol, 1 equiv) was weighed into a Schlenk ampoule then suspended in Et_2O and cooled in an aluminium block placed in the liquid N_2-cooled cold well of the glovebox. After 30 minutes, neopentylMgBr (336 µL, 0.067 mmol, 1 equiv) was added dropwise, resulting in a color change to green. The reaction vessel was removed periodically from the cold well and agitated until all the blue solid **2-Br** seemed to have reacted. The Schlenk was then removed from the glovebox while still in the cold aluminium block, then the procedure to add CO_2 described above was followed.

Catalytic carboxylation reactions

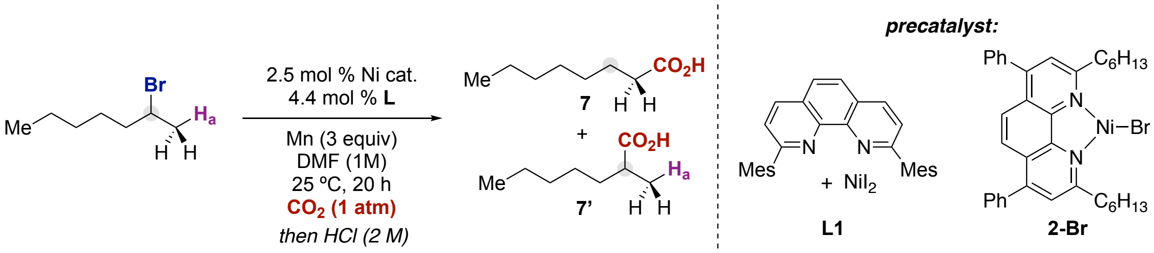

Scheme S2. General scheme for investigating the catalytic ability of 2-Br and L1 complexes.

Following a reported literature procedure:[4]

An oven-dried Teflon-sealed Schlenk ampoule containing a stirring bar was charged with the corresponding Ni source, ligand, and Mn powder reducing agent. If the Ni source was to be [(**L2**)NiBr] (**2-Br**), the Schlenk ampoule was then taken into the glovebox where the **2-Br** was added. Outside the glovebox, the Schlenk tube was evacuated and back-filled under a flow of carbon dioxide (this sequence was repeated three times) then placed under 1 atm CO_2. The solvent and 2-bromoheptane (0.50 mmol) were subsequently added by syringe, then the Schlenk tube was sealed and the solution taken to the corresponding temperature and stirred for 20 h. After this time, the mixture was allowed to equilibrate with room temperature, carefully quenched with 2 M HCl to hydrolyze the resulting Mn-carboxylate, and finally extracted with EtOAc. A sample of the organic layer was analyzed by GC.

Table S1. Screening of 2-Br and L1 complexes in the chain-walking carboxylation of 2-bromoheptane.

Entry	Ni catalyst	L	Conversion (%)[a]	**7** (%)[a]	Ratio **7:7’** [a]
1	NiI_2	**L2**	94	76	98:2
2	NiI_2	**L1**	100	33	99:1
3	[(**L2**)NiBr] (**2-Br**)[b]	**L2**	100	49	97:3

Reaction conditions: 2-bromoheptane (0.50 mmol, 1 equiv), Ni catalyst (2.5 mol %), **L** (4.4 mol %), Mn (1.50 mmol, 3.0 equiv.), CO_2 (1 atm) in DMF (1 M) at 25 ºC for 20 h. [a]Yields and linear/branched ratio were determined by GC FID using anisole as internal standard. [b]When using **2-Br** as precatalyst, 1.9 mol % of **L2** was used instead of 4.4 mol %.

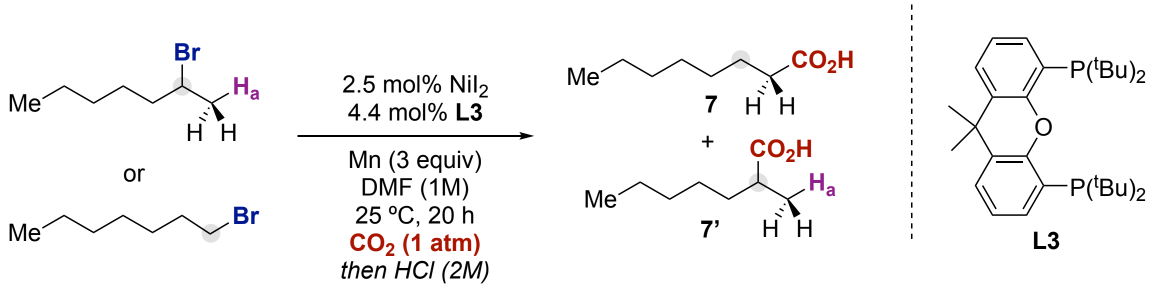

Scheme S3. General scheme for investigating the catalytic ability of L3.

Following a reported literature procedure:[4]

An oven-dried Teflon-sealed Schlenk ampoule containing a stirring bar was charged with the corresponding Ni source, ligand, and Mn powder reducing agent. The Schlenk was evacuated and back-filled under a flow of carbon dioxide (this sequence was repeated three times) then placed under 1 atm CO_2. The solvent and 2-bromoheptane (0.50 mmol) or 1-bromoheptane (0.50 mmol) were subsequently added by syringe, then the Schlenk ampoule was sealed and the solution taken to the corresponding temperature and stirred for 20 h. After this time, the mixture was allowed to equilibrate with room temperature, carefully quenched with 2 M HCl to hydrolyze the resulting Mn-carboxylate, and finally extracted with EtOAc. A sample of the organic layer was analyzed by GC.

Table S2. Screening of L3 in the chain-walking carboxylation of 2-bromoheptane and 1-bromoheptane.

Entry	Substrate	Conversion (%)[a]	**7** (%)[a]	Ratio **7**:**7'** [a]
1	2-bromoheptane	2	0	-
2	1-bromoheptane	1	0	-

Reaction conditions: 1- or 2-bromoheptane (0.50 mmol, 1 equiv), NiI_2 (2.5 mol %), **L3** (4.4 mol %), Mn (1.50 mmol, 3.0 equiv.), CO_2 (1 atm) in DMF (1 M) at 25 °C for 20 h. [a]Yields and linear/branched ratio were determined by GC FID using anisole as internal standard.

IR spectra

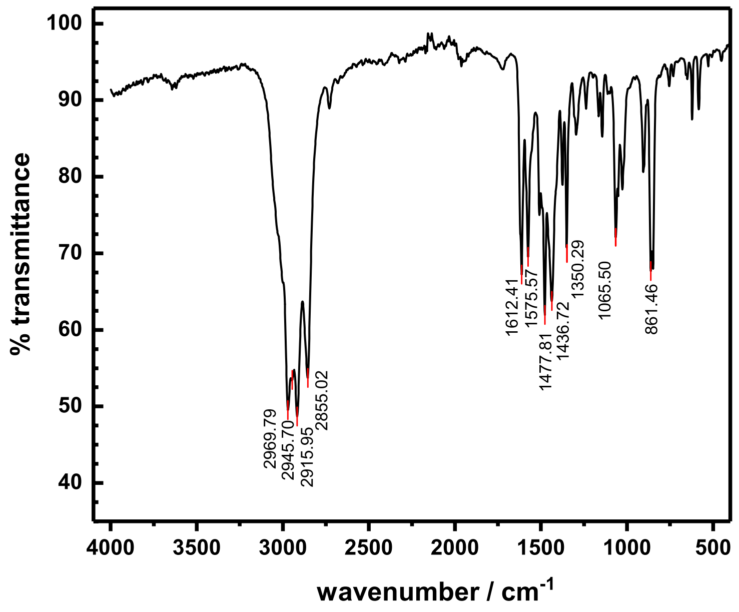

Figure S13. IR spectrum of 1-Br.

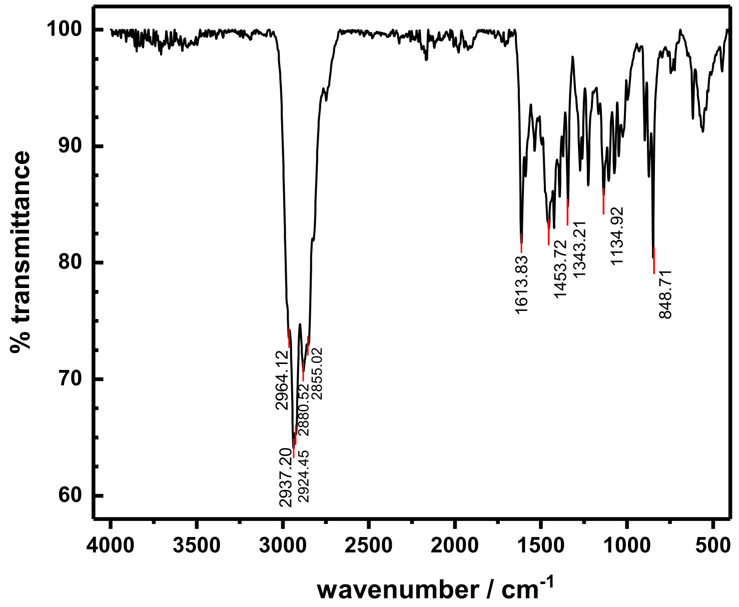

Figure S14. IR spectrum of 3.

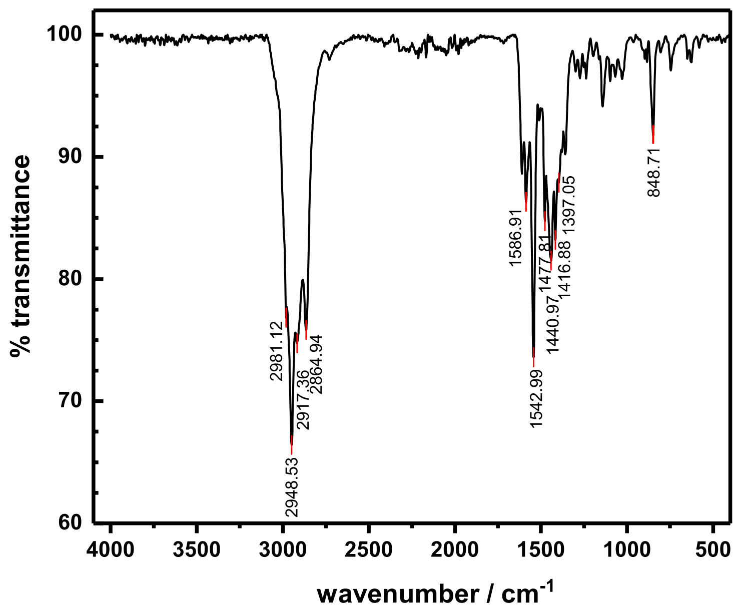

Figure S15. IR spectrum of anion metathesis product (5).

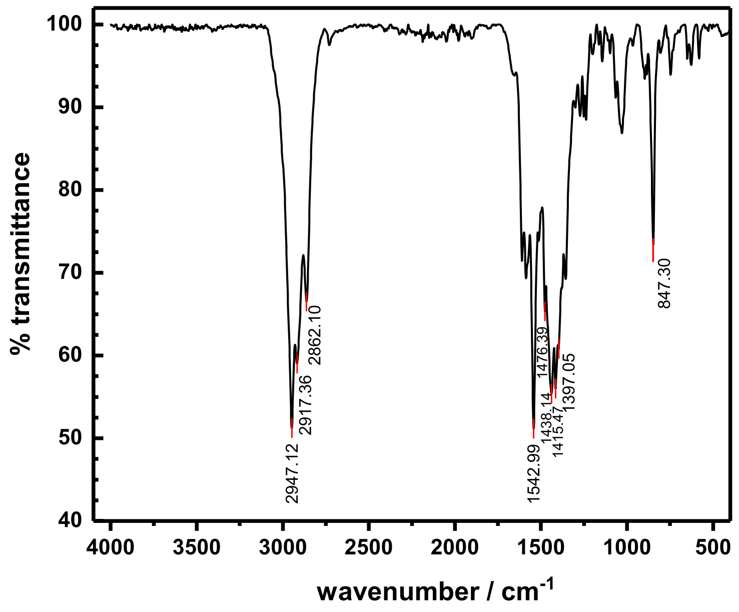

Figure S16. IR spectrum of CO_2 insertion to 3.

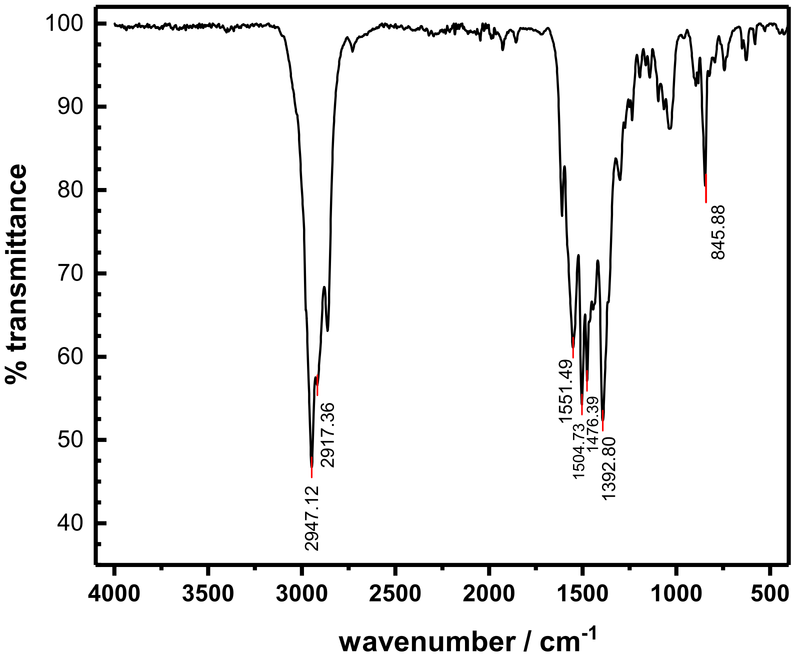

Figure S17. IR spectrum of *$^{13}CO_2$* insertion to 3.

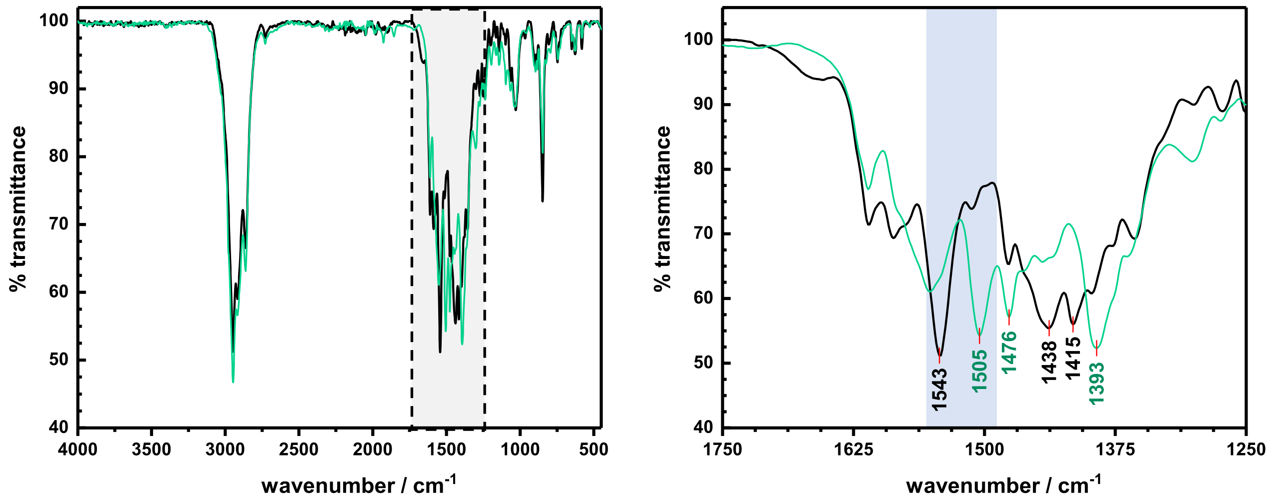

Figure S18. Overlay of IR spectra of CO_2/$^{13}CO_2$ (black/green) insertion to 3.

NMR spectra of halide complexes

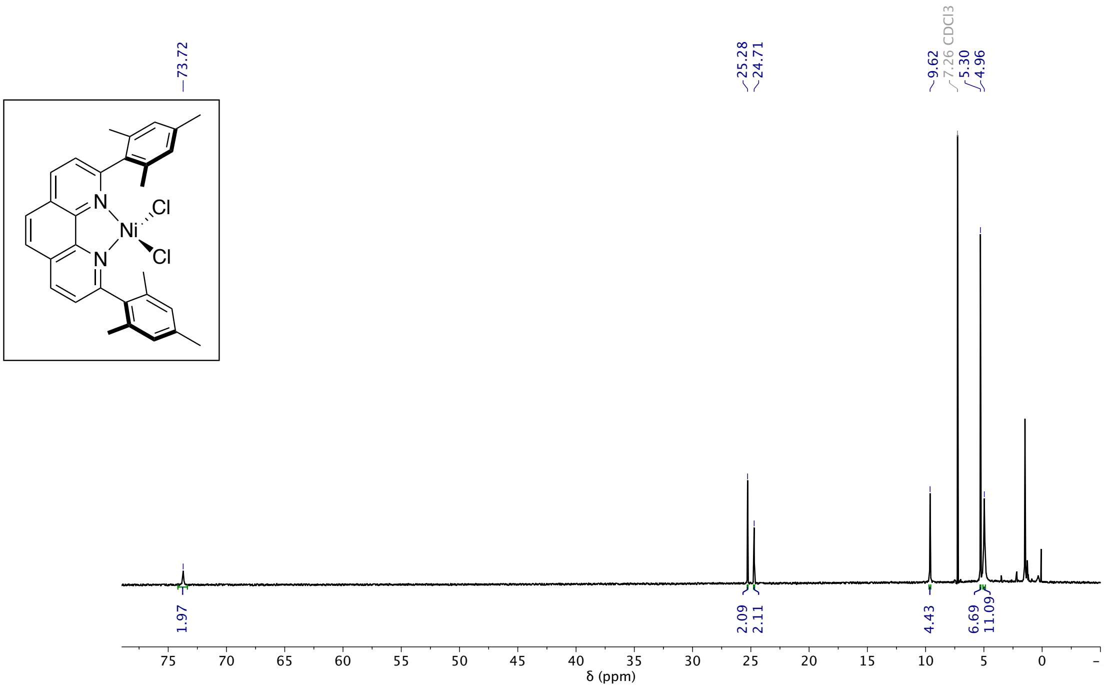

Figure S19. ^{1}H NMR (500 MHz, $CDCl_3$) of [$NiCl_2$(L1)].

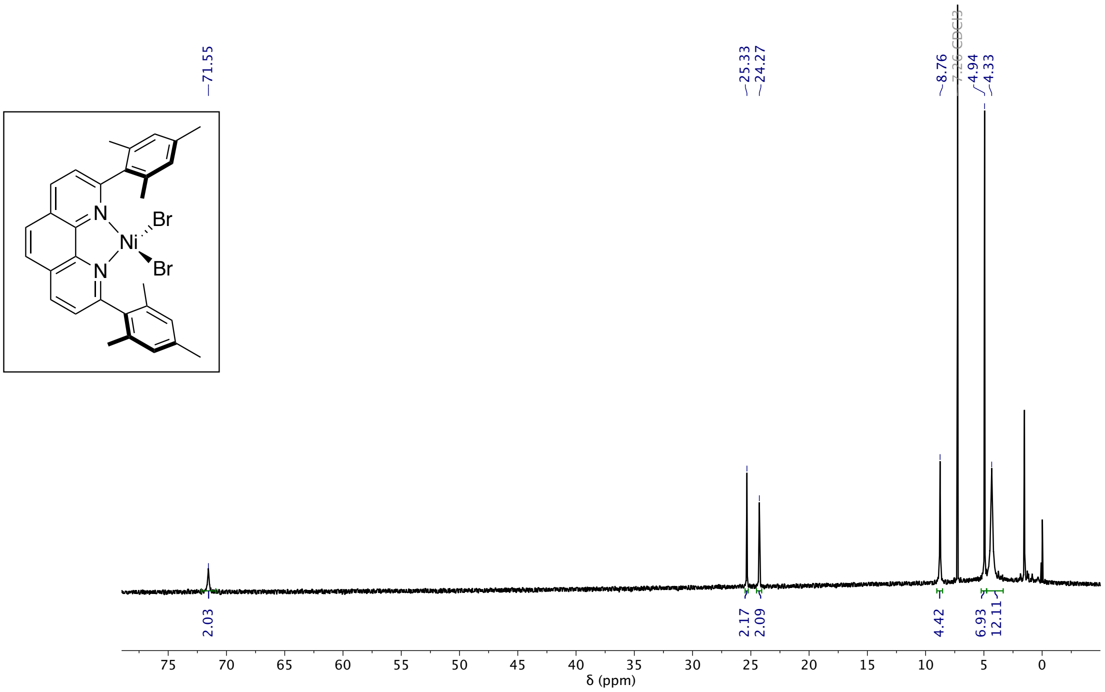

Figure S20. ^{1}H NMR (500 MHz, $CDCl_3$) of [$NiBr_2$(L1)].

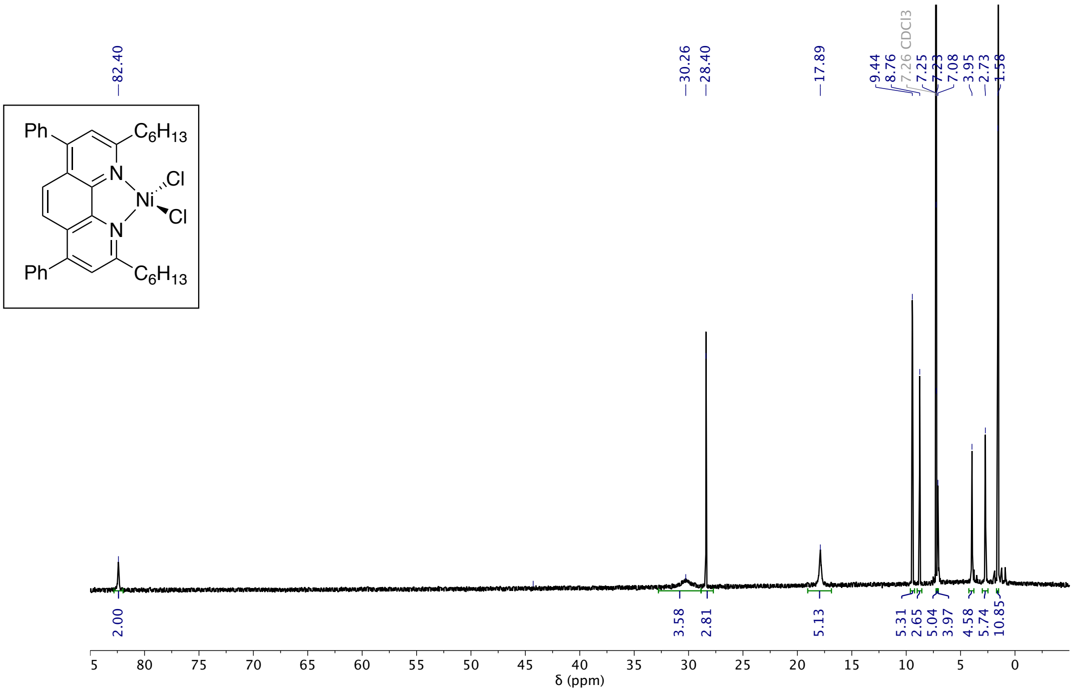

Figure S21. ^{1}H **NMR (400 MHz,** $CDCl_3$**) of** $[NiCl_2(L2)]$**.**

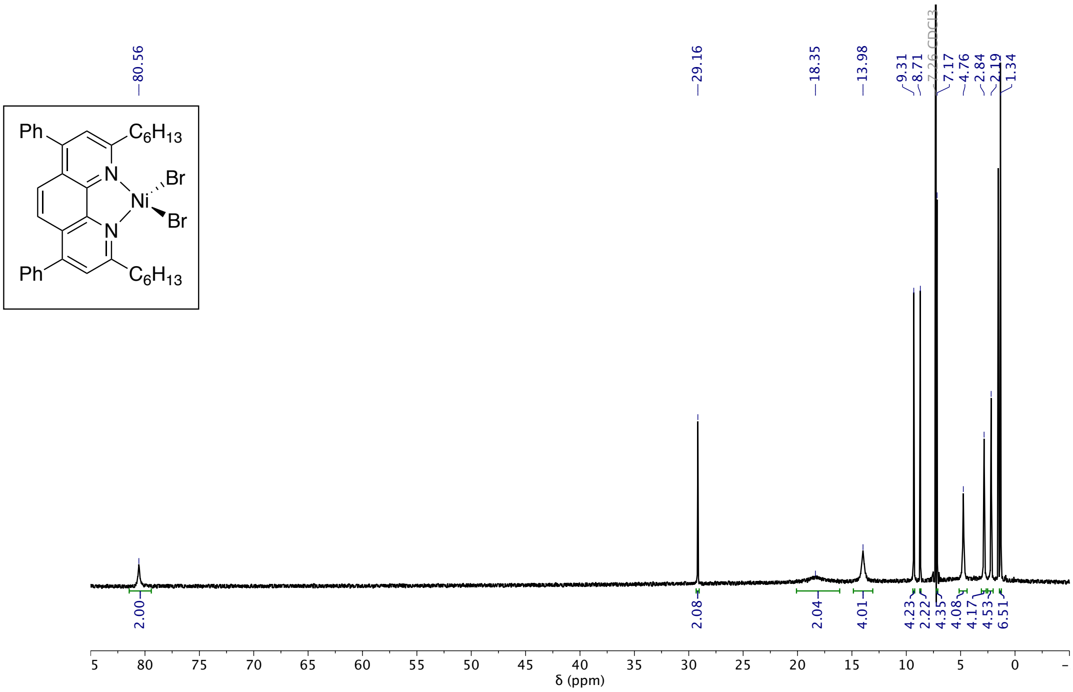

Figure S22. ^{1}H **NMR (400 MHz,** $CDCl_3$**) of** $[NiBr_2(L2)]$**.**

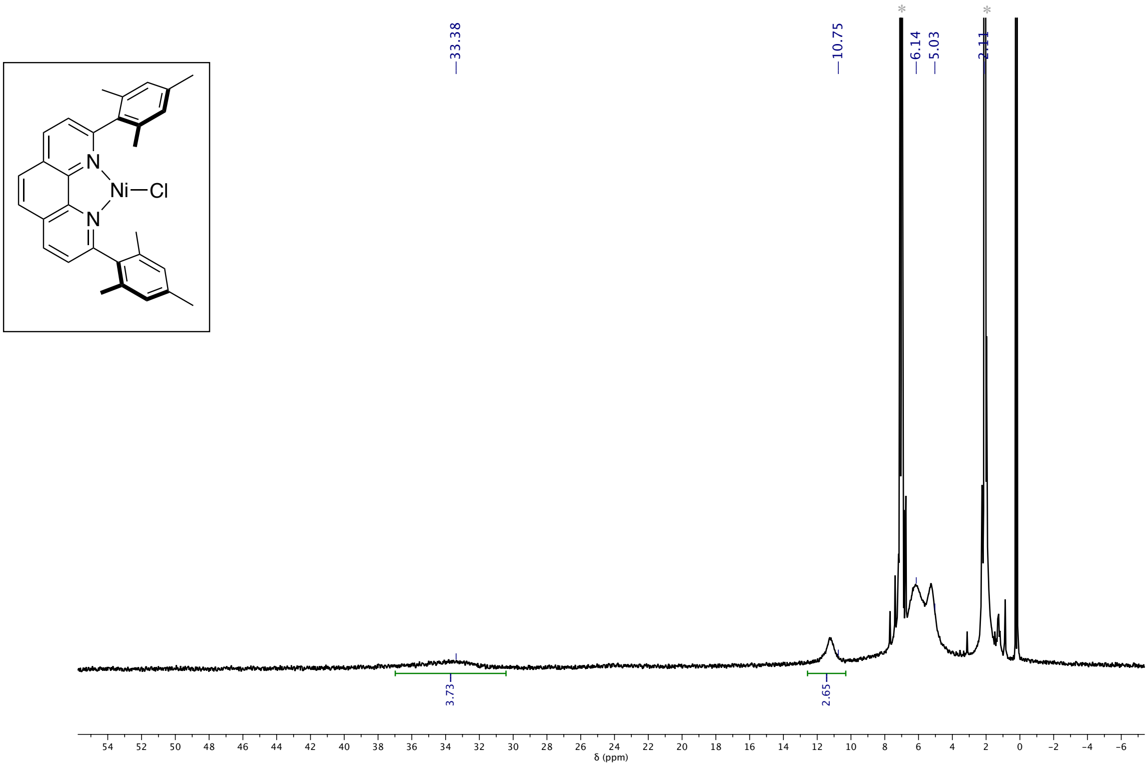

Figure S23. ^{1}H NMR (500 MHz, toluene-d8 (* at 6.98 and 2.08)) of 1-Cl.

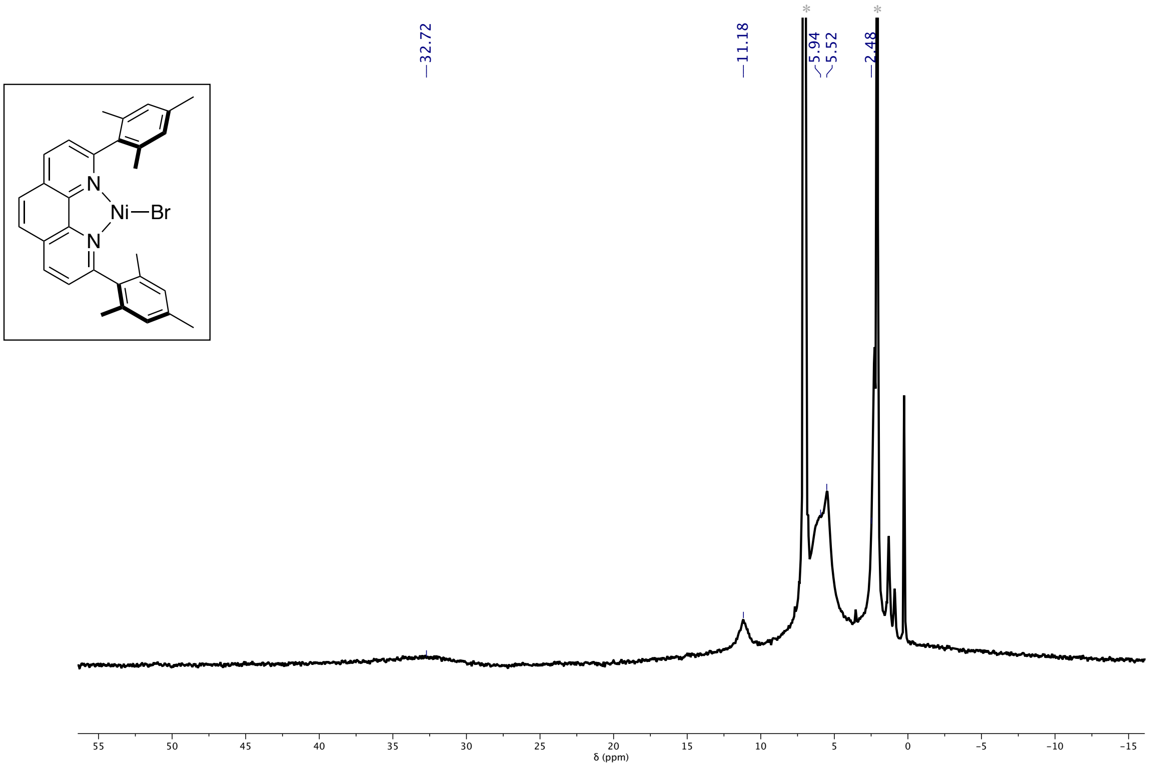

Figure S24. ^{1}H NMR (500 MHz, toluene-d8 (* at 6.98 and 2.08)) of 1-Br.

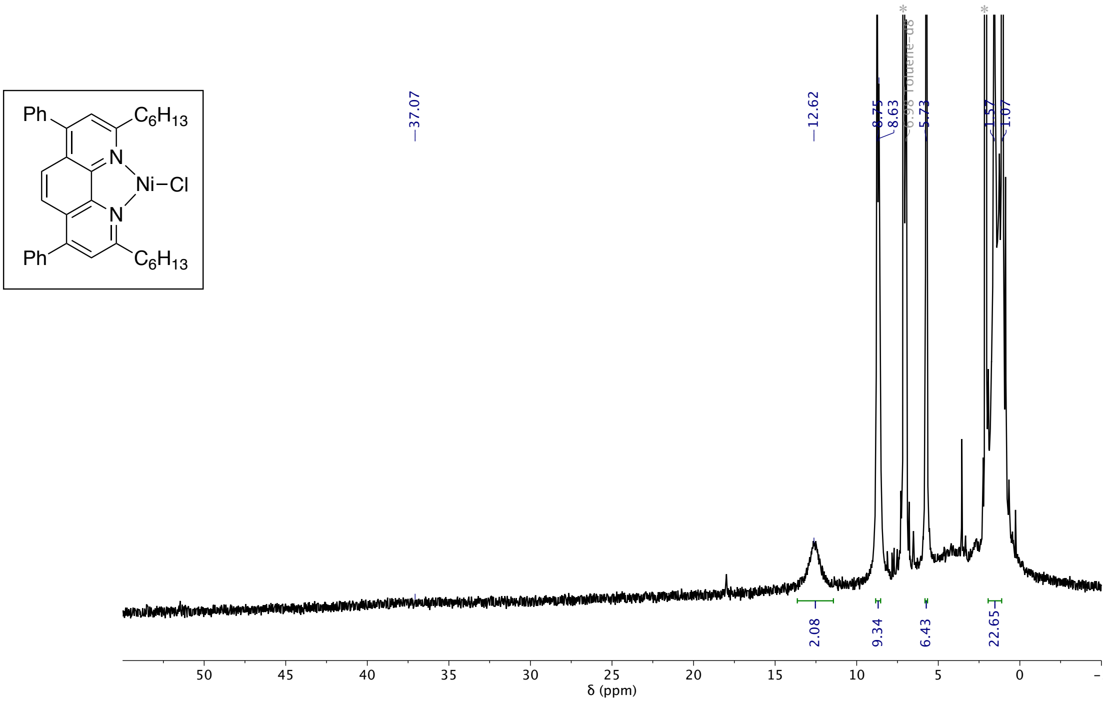

Figure S25. ^{1}H NMR (400 MHz, toluene-d8 (* at 6.98 and 2.08)) of 2-Cl.

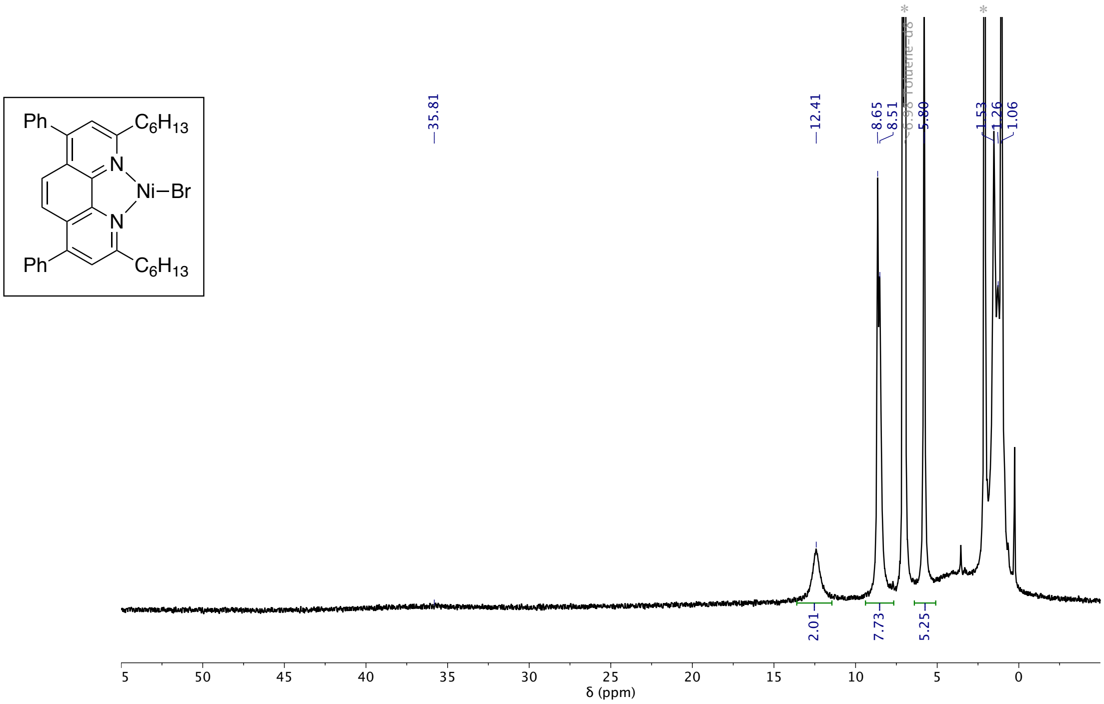

Figure S26. ^{1}H NMR (400 MHz, toluene-d8 (* at 6.98 and 2.08)) of 2-Br.

NMR spectra of alkyl complexes

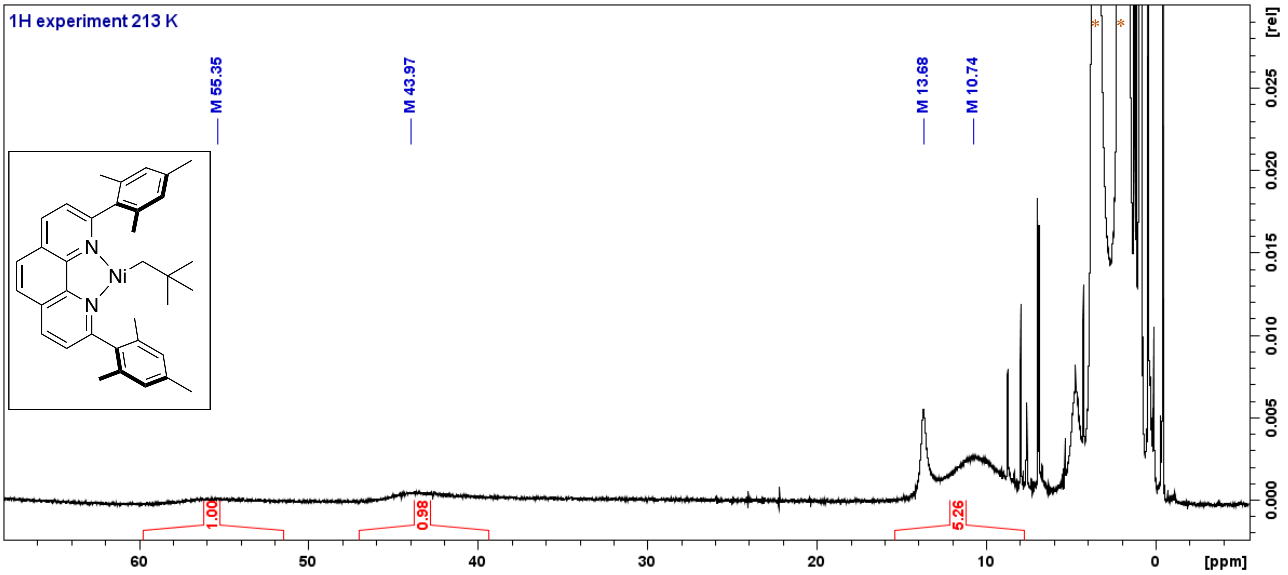

Figure S27. ^{1}H NMR (500 MHz, THF-d_8, 213 K) of 3. Residual solvent peak overlaps with protio-THF of Grignard reagent (*, peaks at ca. 3.6 and 1.7 ppm).

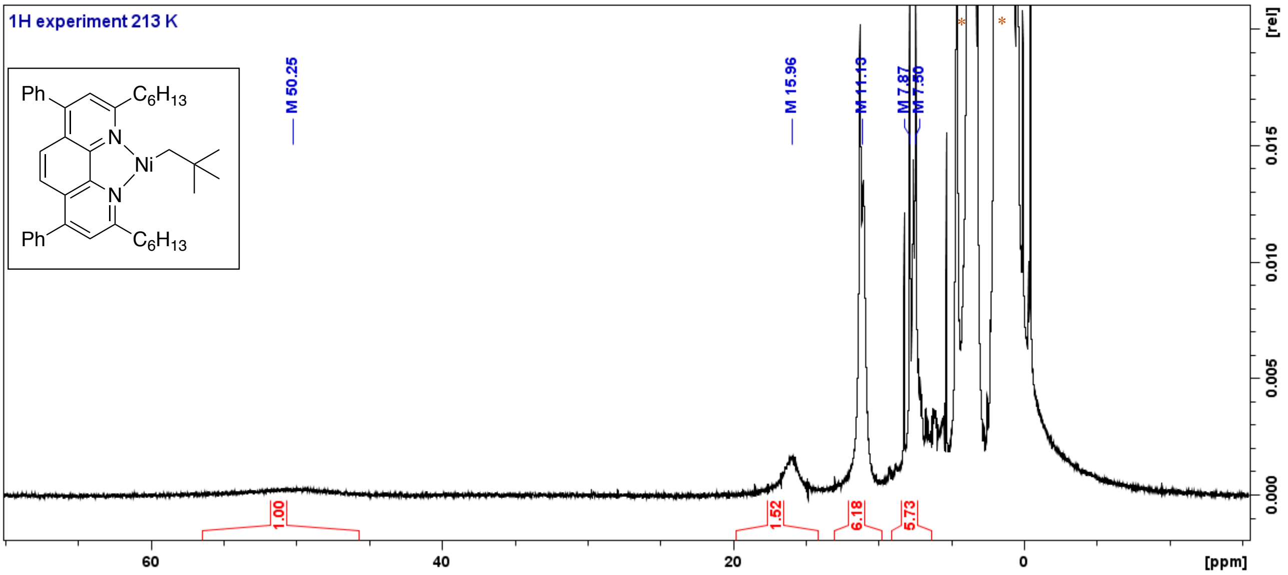

Figure S28. ^{1}H NMR (500 MHz, THF-d_8, 213 K) of 4. Residual solvent peak overlaps with protio-THF of Grignard reagent (*, broad peaks at ca. 3.6 and 1.7 ppm).

NMR spectra of 5

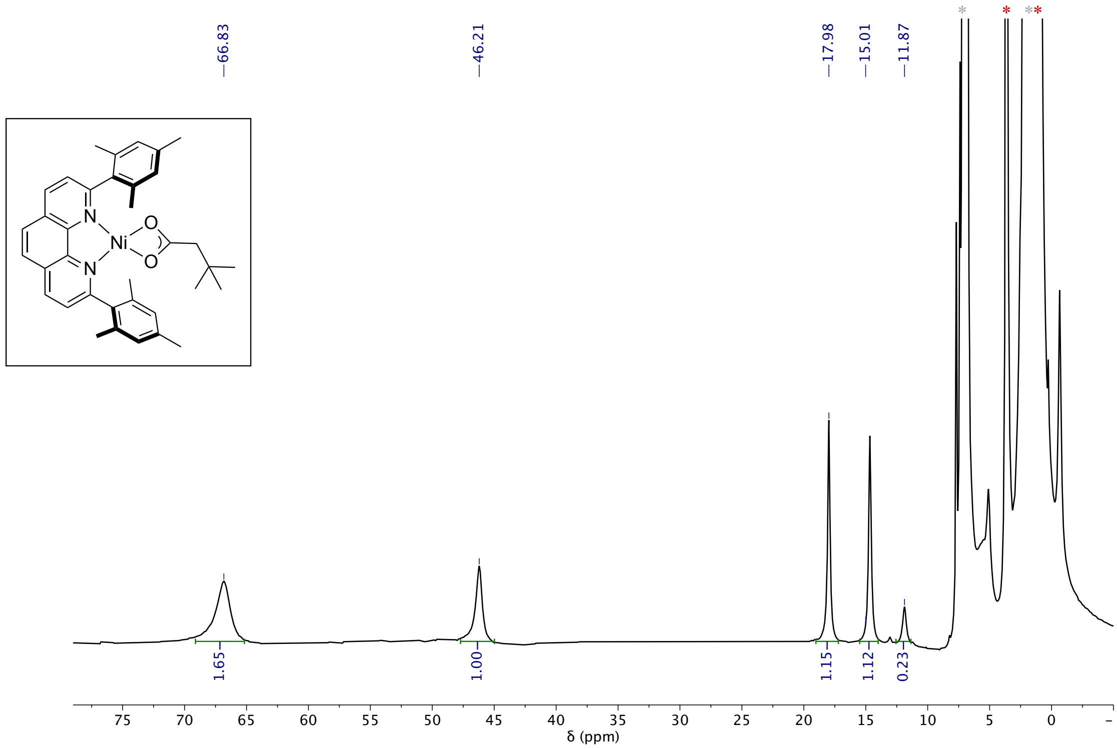

Figure S29. ^{1}H NMR (400 MHz, toluene-d_8 (* at 6.98 and 2.08)) of 5. THF (*)

Crystallographic data

CCDC deposition numbers:
1-Cl: 1990525
2-Cl: 1990348
3: 1990349

Details for 1-Cl (Dr Brandon Q. Mercado, Yale University)

Low-temperature diffraction data (ω-scans) were collected on a Rigaku MicroMax-007HF diffractometer coupled to a Dectris Pilatus3R detector with Mo Kα (λ = 0.71073 Å) for the structure of 007c-18081. The diffraction images were processed and scaled using Rigaku Oxford Diffraction software (CrysAlisPro; Rigaku OD: The Woodlands, TX, 2015). The structure was solved with SHELXT and was refined against F^2 on all data by full-matrix least squares with SHELXL (Sheldrick, G. M. Acta Cryst. 2008, A64, 112–122).

All non-hydrogen atoms were refined anisotropically. Hydrogen atoms were included in the model at geometrically calculated positions and refined using a riding model. The isotropic displacement parameters of all hydrogen atoms were fixed to 1.2 times the U value of the atoms to which they are linked (1.5 times for methyl groups). The program SQUEEZE was used to compensate for the contribution of disordered solvents contained in voids within the crystal lattice from the diffraction intensities. This procedure was applied to the data file and the submitted model is based on the solvent removed data. Based on the total electron density found in the voids (325 e/Å^3), it is likely that ~6.5 toluene molecules are present in the unit cell. See "_platon_squeeze_details" in the .cif for more information.

- Sheldrick, G. M. *SHELXTL* Version 2014/7. http://shelx.uni-ac.gwdg.de/SHELX/index.php

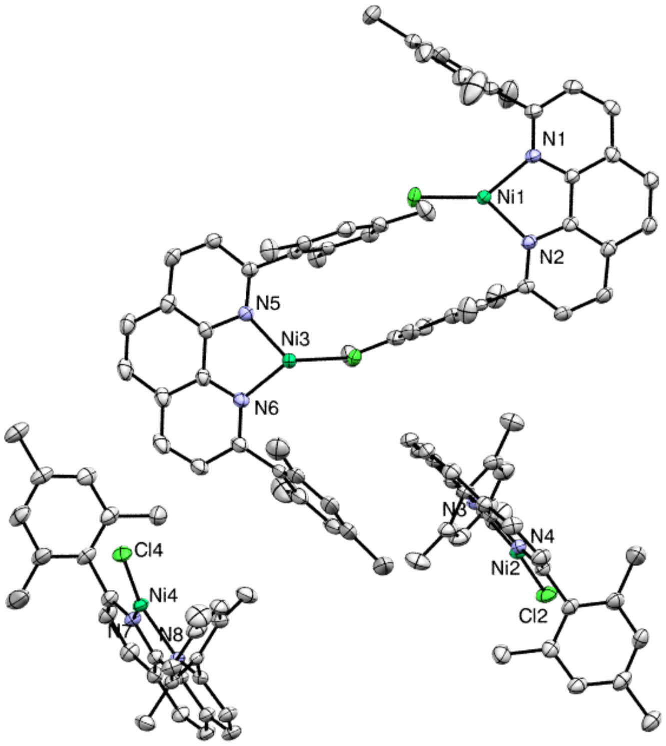

Figure S30. ORTEP diagram of 1-Cl showing the four molecules in asymmetric unit. Thermal ellipsoids drawn at 50% probability, hydrogen atoms hidden for clarity.

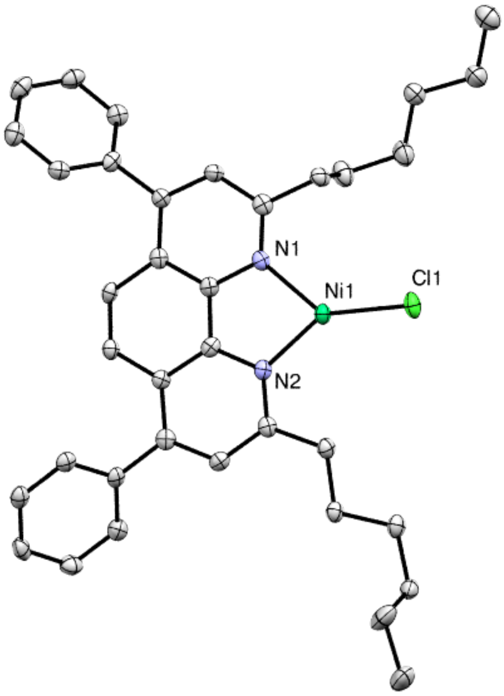

Figure S31. ORTEP diagram of 2-Cl. Thermal ellipsoids drawn at 50% probability, hydrogen atoms hidden for clarity.

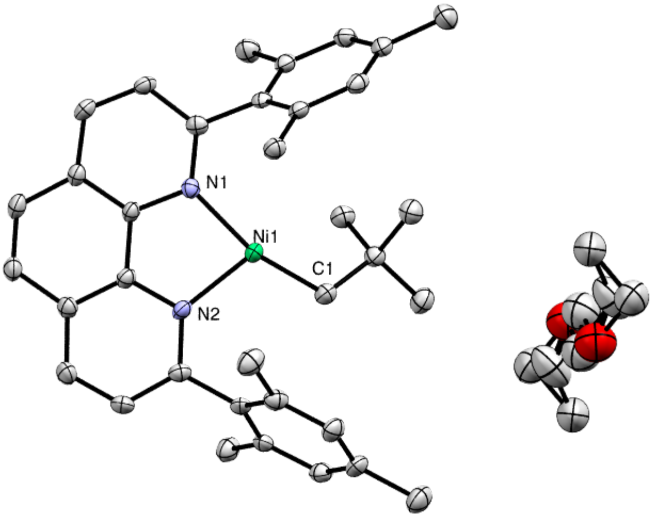

Figure S32. ORTEP diagram of 3 showing disordered diethyl ether (reflected in data shown in Table S3).

See CIF files (included as additional Supporting Information) for full numbering schemes and details.

Table S3. Crystallographic data.

	**1-Cl**	**2-Cl**	**3**
**Formula**	$C_{30}H_{28}ClN_2Ni$	$C_{36}H_{40}ClN_2Ni$	$C_{37}H_{44}N_2NiO_{0.5}$
**Formula weight**	510.70	594.86	583.45
**T (K)**	93(2)	100(2)	100(2)
**Wavelength (Å)**	0.71073	0.71073	0.71073
**Crystal system**	Triclinic	monoclinic	monoclinic
**Space group**	P-1	P 21/c	P 21/c
a **(Å)**	18.0288(15)	9.8891(16)	11.0083(6)
b **(Å)**	18.5080(14)	21.524(3)	32.4592(17)
c **(Å)**	22.4157(15)	14.620(2)	8.8043(5)
**α (deg)**	113.701(7)	90	90
β **(deg)**	90.846(7)	103.029(5)	90.9405(18)
γ **(deg)**	114.996(8)	90	90
**V ($Å^3$)**	6051.7(9)	3031.8(8)	3145.5(3)
**Z**	8	4	4
**Density (calcd.) (Mg/cm^{-3})**	1.121	1.303	1.232
**μ (mm^{-1})**	0.747	0.755	0.646
**F(000)**	2136	1260	1248
**Crystal size (mm^3)**	0.200 x 0.150 x 0.070	0.200 x 0.050 x 0.050	0.300 x 0.050 x 0.020
**Theta range for data collection (deg)**	2.725 to 27.484	2.114 to 26.565	1.954 to 25.076
**Index ranges**	-21<=h<=23, -24<=k<=24, -28<=l<=29	-12<=h<=12, -26<=k<=26, -18<=l<=13	-12<=h<=13, -38<=k<=37, -10<=l<=8
**Reflections collected**	116021	19883	41144
**Independent reflections**	27570 [R(int) = 0.1214]	6057 [R(int) = 0.0687]	5508 [R(int) = 0.1016]
**Completeness to theta**	99.8% (25.242°)	95.5% (26.565º)	98.7% (25.076)
**Absorption correction**	Semi-empirical from equivalents	Multi-scan	Multi-scan
**Max. and min. transmission**	1.00000 and 0.49560	0.74 and 0.49	0.74 and 0.49
**Refinement method**	SHELXL-2014/7 (Sheldrick, 2014)	Full-matrix least-squares on F^2	Full-matrix least-squares on F^2
**Data/restraints/parameters**	27570/0/1249	6057/0/363	5508/61/399
**Goodness-of-fit on F^2**	0.986	1.032	1.055
**Final R indices [I>2sigma(I)]**	R1 = 0.0452, wR2 = 0.1058	R1 = 0.0526, wR2 = 0.1148	R1 = 0.0521, wR2 = 0.1090
**R indices (all data)**	R1 = 0.0799, wR2 = 0.1163	R1 = 0.0935, wR2 = 0.1306	R1 = 0.0821, wR2 = 0.1217
**Largest diff. peak and hole**	0.612 and −0.559 e.$Å^{-3}$	0.845 and −0.554 e.$Å^{-3}$	0.556 and -0.439 e.$Å^{-3}$

Computational Details

All calculations were performed on the full molecular models without any truncations using Gaussian16, Revision B.01.[13] Results in the main text correspond to the GGA functional PBE,[14,15] with additional results using the hybrid functional B3LYP[16] reported in the Supporting Information for comparison. Optimizations with both functionals were done in combination with the Grimme empirical dispersion correction with Becke-Johnson damping D3BJ[17] and the solvent model IEFPCM[18–20](THF). For all calculations, we employed the basis set def2-TZVP. Vibrational mode analysis was performed for all structures to ensure that they have zero (ground state) or exactly one (TS) imaginary frequency. Calculated IR spectra were obtained by utilizing the harmonic approximation, therefore, all obtained wavenumbers/frequencies were scaled according to the CCCBDB[21] (0.986 for PBE, 0.96 for B3LYP) to account for this. All optimized geometries are found in the accompanying .xyz file.

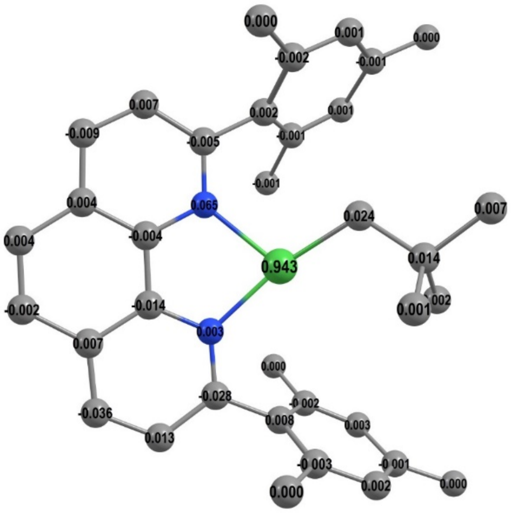

Figure S33. Calculated structure (PBE-D3BJ/def2-TZVP/IEFPCM(THF)) of **3** with the hydrogen atoms removed for clarity. Numbers are computed spin populations. Selected distances (Å) and angles (°): Ni–C 1.981 Å, N1–Ni–C1 159.5°, N2–Ni–C1 108.9°.

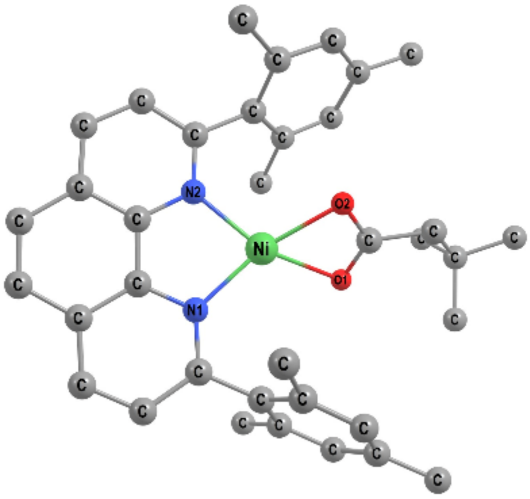

Figure S34. Calculated structure (PBE-D3BJ/def2-TZVP/IEFPCM(THF)) of **5** with the hydrogen atoms removed for clarity. Selected distances (Å) and angles (°): N1/2-Ni 1.99 Å , O1-Ni 2.12 Å , O2-Ni 2.14 Å , N1-Ni-N2 83.2°, O1-Ni-O2 62.6°, N1-Ni-O1 111.4°, N2-Ni-O2 116.5°.

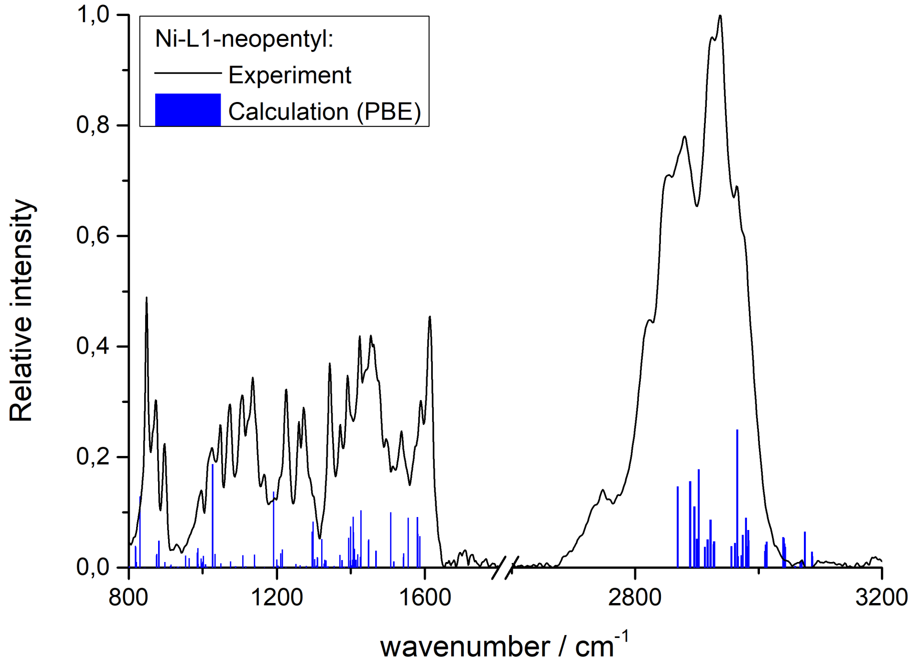

Figure S35. Comparison of the experimental (black) and calculated IR spectra (blue, PBE-D3BJ/def2-TZVP/IEFPCM(THF)) for **3**.

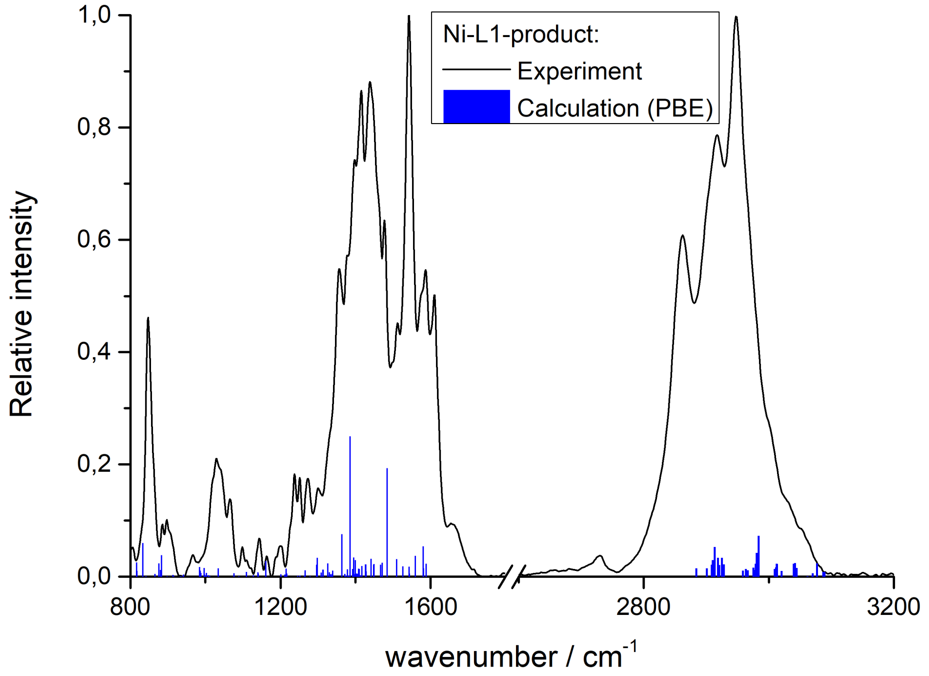

Figure S36. Comparison of experimental (black) and calculated IR spectra (blue, PBE-D3BJ/def2-TZVP/IEFPCM(THF)) for **5**.

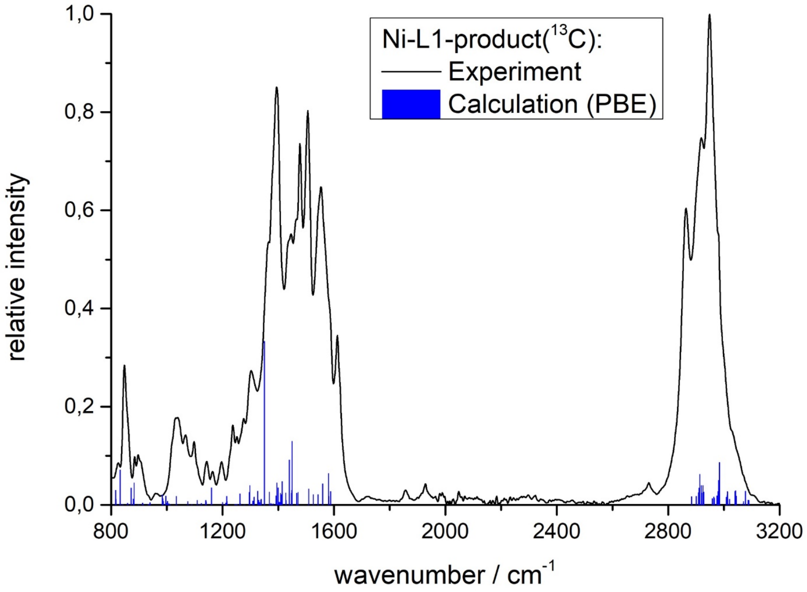

Figure S37. Comparison of experimental (black) and calculated IR spectra (blue, PBE-D3BJ/def2-TZVP/IEFPCM(THF)) for **5**-($^{13}CO_2$).

The influence of replacing the carbon atom of the CO_2 with a ^{13}C atom was computed and predicted a shift of 34 cm^{-1} (1484−1450 cm^{-1}) (PBE-D3BJ) or 36 cm^{-1} (1503−1467 cm^{-1}) (B3LYP-D3BJ) towards lower wavenumbers. This is similar to the experimentally observed shift from 1543 to 1505 cm^{-1} (Δ = 38 cm^{-1}) (see Figure S18) and supports the conclusion that **5** was synthesized.

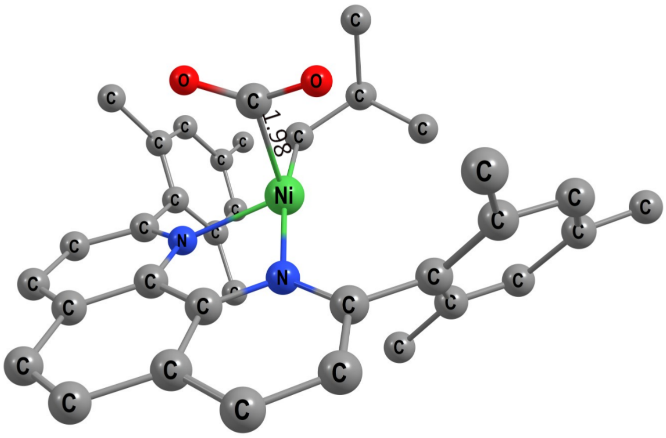

Figure S38. Optimized structure (PBE-D3BJ/def2-TZVP/IEFPCM(THF)) of a potential CO_2-adduct of the Ni(I)-neopentyl complex (**3**). The energy of this species relative to **3** + free CO_2 is 2.8 kcal/mol (7.2 kcal/mol with B3LYP-D3BJ, 298 K). Hydrogen atoms are removed for clarity. Distances are given in Å.

Table S4. Comparison of calculated Gibbs free energy barriers (in kcal/mol, 298K) for CO_2 insertion into 3 with different DFT functionals. Barriers are computed relative to 3 and free CO_2.

Structure (see Figure S34)	PBE-D3BJ	B3LYP-D3BJ
inner sphere TS	7.7	10.4
outer sphere TS	22.7	24.3

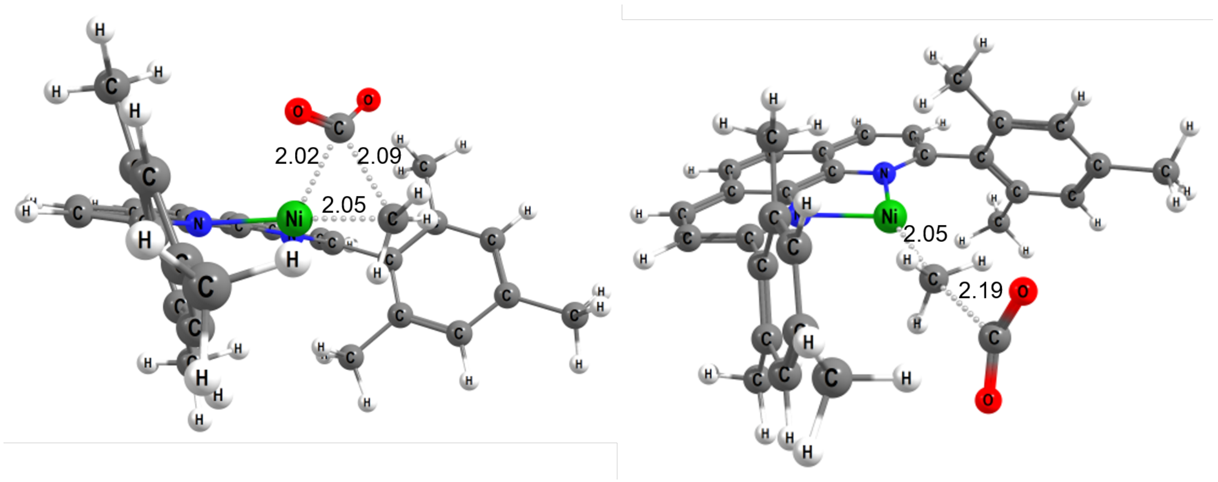

Figure S39. Optimized TS geometries of the *inner* (left) and *outer* sphere (right) CO_2 insertion into (**L1**)Ni(I)Me, which is the methyl analogue of **3**. The *inner* sphere TS is preferred by 6.6 kcal/mol (Gibbs free energy, PBE-D3BJ/def2-TZVP/IEFPCM(THF), 298 K). Distances are given in Å.

References

(1) Torker, S.; Müller, A.; Sigrist, R.; Chen, P. Tuning the Steric Properties of a Metathesis Catalyst for Copolymerization of Norbornene and Cyclooctene toward Complete Alternation. *Organometallics* **2010**, *29* (12), 2735–2751.

(2) Kohler, L.; Hayes, D.; Hong, J.; Carter, T. J.; Shelby, M. L.; Fransted, K. A.; Chen, L. X.; Mulfort, K. L. Synthesis, Structure, Ultrafast Kinetics, and Light-Induced Dynamics of CuHETPHEN Chromophores. *Dalt. Trans.* **2016**, *45* (24), 9871–9883.

(3) Saito, T.; Uchida, Y.; Misono, A.; Yamamoto, A.; Morifuji, K.; Ikeda, S. Diethyldipyridylnickel. Preparation, Characterization, and Reactions. *J. Am. Chem. Soc.* **1966**, *88* (22), 5198–5201.

(4) Juliá-Hernández, F.; Moragas, T.; Cornella, J.; Martin, R. Remote Carboxylation of Halogenated Aliphatic Hydrocarbons with Carbon Dioxide. *Nature* **2017**, *545* (7652), 84–88. https://doi.org/10.1038/nature22316.

(5) Evans, D. F. 400. The Determination of the Paramagnetic Susceptibility of Substances in Solution by Nuclear Magnetic Resonance. *J. Chem. Soc.* **1959**, 2003–2005.

(6) Sur, S. K. Measurement of Magnetic Susceptibility and Magnetic Moment of Paramagnetic Molecules in Solution by High-Field Fourier Trasform NMR Spectroscopy. *J. Magn. Reson.* **1989**, *82*, 169–173.

(7) Meng, Q. Y.; Wang, S.; Huff, G. S.; Konig, B. Ligand-Controlled Regioselective Hydrocarboxylation of Styrenes with CO_2 by Combining Visible Light and Nickel Catalysis. *J. Am. Chem. Soc.* **2018**, *140* (9), 3198–3201.

(8) Hatnean, J. A.; Beck, R.; Borrelli, J. D.; Johnson, S. A. Carbon-Hydrogen Bond Oxidative Addition of Partially Fluorinated Aromatics to a $Ni(P^iPr_3)_2$ Synthon: The Influence of Steric Bulk on the Thermodynamics and Kinetics of C–H Bond Activation. *Organometallics* **2010**, *29* (22), 6077–6091.

(9) Johnson, S. A.; Huff, C. W.; Mustafa, F.; Saliba, M. Unexpected Intermediates and Products in the C−F Bond Activation of Tetrafluorobenzenes with a Bis(Triethylphosphine)Nickel Synthon: Direct Evidence of a Rapid and Reversible C−H Bond Activation by Ni(0). *J. Am. Chem. Soc.* **2008**, *130* (51), 17278–17280. https://doi.org/10.1021/ja8081395.

(10) Li, T.; García, J. J.; Brennessel, W. W.; Jones, W. D. C–CN Bond Activation of Aromatic Nitriles and Fluxionality of the η^2-Arene Intermediates: Experimental and Theoretical Investigations. *Organometallics* **2010**, *29* (11), 2430–2445.

(11) Bach, I.; Pörschke, K.-R.; Goddard, R.; Kopiske, C.; Krüger, C.; Rufińska, A.; Seevogel, K. Synthesis, Structure, and Properties of $\{({}^tBu_2PC_2H_4P{}^tBu_2)Ni\}_2(\mu\text{-}\eta^2{:}\eta^2\text{-}C_6H_6)$ and $({}^tBu_2PC_2H_4P{}^tBu_2)Ni(\eta^2\text{-}C_6F_6)$. *Organometallics* **1996**, *15* (23), 4959–4966.
(12) Johnson, S. A.; Taylor, E. T.; Cruise, S. J. A Combined Experimental and Computational Study of Unexpected C–F Bond Activation Intermediates and Selectivity in the Reaction of Pentafluorobenzene with a $(PEt_3)_2Ni$ Synthon. *Organometallics* **2009**, *28* (13), 3842–3855.
(13) Gaussian 16, Revision B.01, Frisch, M. J.; Trucks, G. W.; Schlegel, H. B.; Scuseria, G. E.; Robb, M. A.; Cheeseman, J. R.; Scalmani, G.; Barone, V.; Petersson, G. A.; Nakatsuji, H.; Li, X.; Caricato, M.; Marenich, A. V.; Bloino, J.; Janesko, B. G.; Gomperts, R.; Mennucci, B.; Hratchian, H. P.; Ortiz, J. V.; Izmaylov, A. F.; Sonnenberg, J. L.; Williams-Young, D.; Ding, F.; Lipparini, F.; Egidi, F.; Goings, J.; Peng, B.; Petrone, A.; Henderson, T.; Ranasinghe, D.; Zakrzewski, V. G.; Gao, J.; Rega, N.; Zheng, G.; Liang, W.; Hada, M.; Ehara, M.; Toyota, K.; Fukuda, R.; Hasegawa, J.; Ishida, M.; Nakajima, T.; Honda, Y.; Kitao, O.; Nakai, H.; Vreven, T.; Throssell, K.; Montgomery, J. A., Jr.; Peralta, J. E.; Ogliaro, F.; Bearpark, M. J.; Heyd, J. J.; Brothers, E. N.; Kudin, K. N.; Staroverov, V. N.; Keith, T. A.; Kobayashi, R.; Normand, J.; Raghavachari, K.; Rendell, A. P.; Burant, J. C.; Iyengar, S. S.; Tomasi, J.; Cossi, M.; Millam, J. M.; Klene, M.; Adamo, C.; Cammi, R.; Ochterski, J. W.; Martin, R. L.; Morokuma, K.; Farkas, O.; Foresman, J. B.; Fox, D. J. Gaussian, Inc., Wallingford CT, **2016**.
(14) Perdew, J. P.; Burke, K.; Ernzerhof, M. Generalized Gradient Approximation Made Simple. *Phys. Rev. Lett.* **1996**, *77* (18), 3865.
(15) Perdew, J. P.; Burke, K.; Ernzerhof, M. Generalized Gradient Approximation Made Simple [Phys. Rev. Lett. 77, 3865 (1996)]. *Phys. Rev. Lett.* **1997**, *78* (7), 1396.
(16) Becke, A. D. Density-Functional Thermochemistry. III. The Role of Exact Exchange. *J. Chem. Phys.* **1993**, *98* (5), 645–648.
(17) Grimme, S.; Ehrlich, S.; Goerigk, L. Effect of the Damping Function in Dispersion Corrected Density Functional Theory. *J. Comput. Chem.* **2011**, *32* (7), 1456–1465.
(18) Tomasi, J.; Mennucci, B.; Cammi, R. Quantum Mechanical Continuum Solvation Models. *Chem. Rev.* **2005**, *105* (8), 2999–3094.
(19) Tomasi, J.; Mennucci, B.; Cances, E. The IEF Version of the PCM Solvation Method: An Overview of a New Method Addressed to Study Molecular Solutes at the QM Ab Initio Level. *J. Mol. Struct. THEOCHEM* **1999**, *464* (1–3), 211–226.
(20) Cances, E.; Mennucci, B.; Tomasi, J. A New Integral Equation Formalism for the Polarizable Continuum Model: Theoretical Background and Applications to Isotropic and Anisotropic Dielectrics. *J. Chem. Phys.* **1997**, *107* (8), 3032–3041.
(21) Computational Chemistry Comparison and Benchmark DataBase, National Institute of Standards and Technology (NIST) cccbdb.nist.gov/vibscalejust.asp (accessed Jan 10, 2019).
